# Serglycin’s role in primary liver cancer: insights into tumor microenvironment and macrophage interaction

**DOI:** 10.3389/fimmu.2025.1668627

**Published:** 2025-12-16

**Authors:** Qinghai Lian, Chonghan Ma, Xiaoxiao Wang, Jiani Wang, Jindi Zeng, Jiongshan Zhang, Yongwei Li

**Affiliations:** 1 Vaccine Research Institute, Sun Yat-Sen University, Guangzhou, Guangdong, China; 2 Cell-Gene Therapy Translational Medicine Research Centre, The Third Affiliated Hospital, Sun Yat-Sen University, Guangzhou, Guangdong, China; 3 Biotherapy Center, The Third Affiliated Hospital, Sun Yat-Sen University, Guangzhou, Guangdong, China; 4 Department of Traditional Chinese Medicine, The Third Affiliated Hospital of Sun Yat-sen University, Guangzhou, Guangdong, China; 5 Department of Thyroid and Breast Surgery, The Third Affiliated Hospital of Sun Yat-sen University, Guangzhou, Guangdong, China; 6 Department of Stomatology, First Affiliated Hospital of Guangzhou Medical University, Guangzhou, Guangdong, China

**Keywords:** serglycin, primary liver cancer, single cell, prognosis, pro-tumorigenicity

## Abstract

**Background:**

Serglycin (SRGN) is an important proteoglycan that regulates tumorigenesis, but its role in primary liver cancer (PLC) remains unclear.

**Methods:**

We investigated the expression and prognostic potential of SRGN in PLC using bioinformatics analyses. HepG2 cells were transfected with an SRGN over expression vector and their proliferation, migration, invasion, resistance to sorafenib, and angiogenic capacity were examined *in vitro*. A subcutaneous xenograft tumor model was created using nude mice. SRGN overexpressing hepatoma cells were co-cultured with THP-1 derived macrophages. The expressions of CD80 and CD206, secretory molecules, and the NF-κB and STAT3 signal pathways were examined by flow cytometry, ELISA and western blot, respectively. Transwell migration and invasion were investigated in HepG2 and Huh7 co-cultured with SRGN-promoted macrophages.

**Results:**

Single-cell analysis revealed SRGN expression across 17 distinct cell subpopulations, with higher expression in macrophages in tumor tissues compared to those in normal tissues. SRGN displayed consistent high expression across cell cycle phases while exhibited dynamic expression during macrophage pseudotime trajectory. Cell communication analysis indicated that SRGN was involved in interactions within the tumor microenvironment (TME), particularly in the VEGF signaling network. Autocrine SRGN promoted *in vitro* aggressiveness, especially pro-angiogenic activity, and *in vivo* tumorigenicity of HepG2 cells, and conferred resistance to sorafenib. Paracrine SRGN promoted a partial polarization of TAMs toward an M2-like phenotype, accompanied by the activation of signaling pathways including NF-κB and STAT3. Levels of secreted argase1 and SRGN were increased in the supernatant. The invasion and migration of hepatoma cells were promoted by SRGN-overexpressing TAMs.

**Conclusions:**

Our findings highlight the role of SRGN in the TME of PLC. SRGN-high TAMs are induced by paracrine SRGN from hepatoma cells, establishing a self-reinforcing mechanism that drives PLC progression. Therapeutic strategies targeting SRGN should take into account its context-specific roles depending on TME cells.

## 1 Introduction

Hepatocellular carcinoma (HCC), accounting for approximately 90% of primary liver cancer (PLC), represents the fourth leading cause of cancer-related mortality globally (1, 2). PLC demonstrates significant heterogeneity across various dimensions, including diverse etiologies and molecular subgroups (3). A key etiological factor is hepatitis B virus (HBV) infection, which alone affects an estimated 250 million individuals worldwide (4, 5). The TME of PLC is highly complex, comprising immunosuppressive and inflammatory cytokines, along with various immune cells like macrophages, neutrophils, and lymphocytes. Despite the emergence of immunotherapy with programmed cell death 1 protein 1 (PD-1)/programmed cell death ligand 1 (PD-L1) blockade as a first-line treatment, its efficacy remains limited, underscoring the necessity for a deeper understanding of the mechanisms linking malignancy and immunity to develop novel therapeutic approaches (6, 7).

Serglycin (SRGN) is a prominent hematopoietic proteoglycan. Its core protein consists of eight serine-glycine repeats that are modified by variable glycosaminoglycan side chains, depending on the cell type and status (8, 9). The function of SRGN is to interact with proteases, chemokines, and cytokines, and is required for the formation of secretory granules. It has been studied in various immune cells—including neutrophils, macrophages, CD8+ T cells, etc., where it helps maintain immune cell population homeostasis by controlling the magnitude and durability of immune responses (10–15). Immunohistochemical studies reveal elevated SRGN expression in advanced tumors and activated tumor microenvironment (TME) across various cancers (16, 17). It exerts pro-tumorigenic effects in multiple ways (18, 19). However, the role of SRGN in cancer remains controversial. Serglycin is highly expressed by infiltrating immune cells in breast cancer(BC) tissues, while the mRNA and protein levels of SRGN were overexpressed in lung adenocarcinoma (LUAD) cell lines, *in vivo* accompanied by higher expression of PD-L1 in cancer cells and higher infiltration of PD-1^+^lymphocytes (20, 21). SRGN was associated with poor outcomes for both BC and LUAD, but with favorable prognosis for skin cutaneous melanoma. In an autocrine manner, SRGN plays the pro-tumorigenic role in several studies (22, 23). Despite the properties of SRGN in both immunity and malignancy (10, 18, 24–26), its specific role within the TME remains incompletely studied through a paracrine signaling pathway.

Our prior work demonstrated elevated hematopoietic SRGN levels in HBV-related HCC patients vs. healthy controls, contrasting with reduced SRGN mRNA in HBV-integrated HepG2.215 cells compared to parental HepG2 (27, 28). IHC confirms SRGN protein overexpression in 56.7% HCC specimens compared to 3.1% non-tumor counterparts (10). SRGN expression in both hematopoietic cells and tumor tissues predicted poor patient outcomes. This study aims to investigate the expression and prognostic value of SRGN using bioinformatics and further explore its role using *in vitro* and *in vivo* models. We focus on elucidating the underlying mechanisms of SRGN in PLC, particularly its interactions with tumor-associated macrophages (TAMs) in the TME. Our findings collectively highlight SRGN as a potential therapeutic target in PLC.

## 2 Materials and methods

### 2.1 Single-cell RNA-seq data analysis workflow

An integrated analysis was conducted on the hepatocellular carcinoma single-cell RNA sequencing dataset GSE242889. For quality control, cells were excluded if they met any of the following criteria: detection of fewer than 200 genes, detection of more than 5,000 genes (indicative of potential doublets), or mitochondrial gene content exceeding 10% of total counts. The Seurat package was employed for data normalization, dimensionality reduction, and clustering. Data normalization, dimensionality reduction, and clustering were performed using the Seurat package. To address batch effects, correction was applied to the top 2,000 most variable genes with the Harmony package under default parameters. Cell clustering and dimensionality reduction were carried out using the ‘FindClusters’ and ‘RunUMAP’ functions, respectively, with the resolution parameter optimized to 0.3 through systematic evaluation. Cell type annotation was achieved by manual mapping against liver-specific marker genes obtained from the ACT database (http://xteam.xbio.top/ACT/index.jsp) (29, 30), and supplemented by automated scoring via the UCell algorithm based on cell-type signature gene sets (31). Interactions among cell types were analyzed with CellChat, focusing on receptor-ligand interactions as defined in the database, using default parameters. Cell cycle scoring was performed with the CellCycleScoring function in Seurat, which calculates S-phase and G2/M-phase scores from predefined gene sets and assigns each cell to G1, S, or G2M phase based on phase-specific marker expression. The resulting scores and phase classifications were stored in the metadata slot for subsequent analyses.

### 2.2 Differentially expressed gene analysis

The expression data, represented as HTseq-Counts, were categorized into high and low expression cohorts based on the median expression level of SRGN. Subsequently, these groups underwent further analysis utilizing the unpaired Student’s t-test within the DESeq2 R package (version 1.36.0). A significance threshold was established, with adjusted p-values <0.05 and |log2-fold change (FC)| >1 deemed as indicators for DEGs. Gene Set Enrichment Analysis (GSEA) was performed using the clusterProfiler package to further explore the biological functions and pathways associated with the DEGs (32, 33). Before GSEA, gene ID conversion was conducted to ensure the compatibility of gene identifiers with the gene sets in the MSigDB collections database.

### 2.3 Immune cell infiltration

Immune microenvironment characterization was performed using single-sample gene set enrichment analysis (ssGSEA) implemented via the GSVA package (34), utilizing 24 gene sets corresponding to immune cell signatures (35). These gene sets enabled the calculation of immune infiltration levels for each sample. Gene-immunocyte correlations were assessed using Spearman’s rank correlation, and the results were visualized as lollipop plots using the ggplot2 package. Additionally, we referred to previous studies (31, 32) to identify specific immune checkpoint genes. The correlation between SRGN and these immune checkpoint genes was analyzed using Spearman’s rank correlation, and the results were visualized using a rose diagram (36).

### 2.4 SRGN expression, prognosis analysis and correlation analysis

The differential expression and prognosis of SRGN in pan-cancer were studied using Gene Expression Profi;ling Interactive Analysis (GEPIA, http://gepia.cancer-pku.cn/index.html) (37), Kaplan–Meier plotter databases (http://kmplot.com/analysis/(registration-freeKM-plotter)) (38), Tumor Immune Estimation Resource (TIMER, https://cistrome.shinyapps.io/timer/) (39) Clinical Proteomic Tumor Analysis Consortium (CPTAC, https://proteomics.cancer.gov/programs/cptac) (40) or Human Protein Atlas (HPA, https://www.proteinatlas.org/) (41). The correlations between SRGN and TME cells or gene markers were studied by Spearman’s correlation analysis via TIMER which was determined using the following criteria: 0.1–0.3, weak correlation, 0.3–0.5, moderate correlation, and 0.5–1.0, strong correlation (42).

### 2.5 Over-expression of SRGN in HepG2 cells

SRGN sequences were designed following the gene (Homo sapiens (human) Gene, variant 3, NM, NCBI132 Reference 105 Sequence 3.2, mRNA, NCBI132 ID: 5552). SRGN XhoI F: 5’ccgctcgag ccaccATGATGCAGAAGCTACTCAAATGCAGTC3’, BamHI R: 5’cgcggatccTTATAACATAAAATCC TCTTCTAATCCATG 3’. RNA was extracted by Trizol, amplificated by PCR. SRGN and pLVX- IRES-Neo vector 15μL each was digested with XhoI/BamHI, purified and ligated. The product was transformed to DH5α competent cells. The positive clones were cloned into pLVX-IRES-Neo vector. The shuttle plasmid containing the target sequence along with the packaging plasmids pVSV-G, pRev, and pGag/Pol were constructed and prepared by Guangzhou Yeshan Biotechnology Co., Ltd., and 293T cell was co-transfected with the transfection reagent LipofectamineTM 2000. After 72 hours of culture, the lentiviral particles were collected and infected HepG2 cells with 400μg/mL G418 to screen for one month. HepG2 cells stably overexpressing SRGN or a blank pLVX-IRES-Neo vector were constructed, and named HepG2SG and HepG2-NC, respectively.

### 2.6 Real-time quantitative polymerase chain reaction

The primers were as follows: SRGN 150 bp, F: CTGCAAACTGCCTTGAAGAA, R: GTGGGAA GATACGATTCAAGTC; β-actin 275 bp, F: TGGATCAGCAAGCAGGAGTA, R: TCGGCCACATT GTGAACTTT. qPCR assays were performed in HepG2-NC group and HepG2SG. Following the manufacturer’s instructions, RNA was extracted with Trizol (Invitrogen Corp, Carlsbad, CA, USA), and cDNA was synthesized with a first-strand cDNA synthesis kit (Takara Inc., Dalian, P. R. China). SYBR Green qPCR SuperMix (Invitrogen Corp) was used for qPCR (ABI, PRISM^®^ 7500 Sequence Detection System). The reaction conditions were 50°C 2 min, 95°C 2 min, 95°C 15 s, 60°C 32 s, 40 cycles, melting curve analysis at 60°C–95°C. The independent experiment was repeated in triplicate, similarly for the following methods. Relative mRNA expression was analyzed by the 2^−△△Ct^ method (43). Cell-cycle qPCR: HepG2-NC and HepG2SG cells were incubated with 10 μg/mL Hoechst-33342 (37 °C, 20 min), and G1/S/G2/M populations were sorted on a BECKMAN COULTER CytoFLEX STR (≥ 1 × 10^5^ cells/phase). RNA was extracted immediately, reverse-transcribed, and SRGN mRNA quantified as above.

### 2.7 Western blot

The proteins were extracted in RIPA buffer (Thermo Scientific™, USA), quantified by the bicinchoninic acid assay, separated by 12% SDS-PAGE, and transferred to polyvinylidene difluoride membranes (Immobilon-P Transfer Membrane, Millipore, IPVH00010). After blocking in TBST buffer, membranes were incubated with Rabbit Anti-SRGN antibody (bs-6789R, BIOSS, Beijing, China ratio 1:1000), Anti-NF-κB(D14E12, CST, Danvers, MA, USA)and p65 antibody (Ser468, #8242 and #3039, CST), Phospho-Stat3 (Ser727, #9134, CST), and Anti-STAT3 antibody (EPR787Y, ab68153, Abcam, Boston, MA, USA) with a GAPDH antibody serving as the loading control (Catalog No.KC-5G5, Kangcheng Bio, Shanghai, China, ratio 1:10,000). After incubation with peroxidase-labeled rabbit anti-rat IgG (Bode Biotech Co., Ltd., Wuhan, China, Cat. no BA1058) secondary antibody at 1:2000 and 37 °C for 1 h, the bands were read with a Pro-light HRP Chemiluminescence Kit (TIANGEN, Beijing, China), and Image J software (Gel Image Analyzer, Tianneng Technology Co., Ltd, Shanghai, China, Tanon 1220) (44).

### 2.8 CCK8 assay of cell viability

For the cell viability assay, the cell cultures were allocated to a HepG2-NC group and HepG2SG groups, and treated with 2, 5, 10, 15, 20, 25μm sorafenib (S7397, Selleck, Houston, TX, USA) diluted in DMSO (S1209, Selleck), respectively. Cell viability was evaluated by a CCK8 assay (CellTiter 96 AQueous One Solution Cell Proliferation Assay, Cat. No. G3582, Promega, Madison, USA). The cell density was 1×10^4^ cells/100μL per well into a 96-well plate. The cells were collected at each time point (0, 1, 3, and 5 days), and 10 μL CCK-8 solution was added according to the manufacturer’s instructions. The results were obtained with five replicates each and read at an absorbance of 490 nm using multiscan MK3 plate reader (Thermo Fisher Scientific Ltd, Waltham, MA, USA). The effect on cell viability at each assay time was reported as Proliferation rate= (mean OD value at time point÷ mean OD value at primary time point-1) ×100%.

### 2.9 Transwell invasion assay

Invasiveness was assayed in HepG2-NC and HepG2SG groups. The methods were performed as before (45). The results were read at absorbance of 570 nm using multiscan MK3 plate reader.

### 2.10 Angiogenesis experiment and vasculogenic mimicry

The Matrigel (BD Company, 356234, USA) was added 150-200uL to each well of 48-well plates, and solidified at 37°°C for more than 2h. Human umbilical vein endothelial cells (HUVECs) were incubated with serum-free medium of respective HepG2SG and HepG2-NC for 12 h, then added 2×10^4^ cells/200uL medium to each well, the tube formation was observed after 4-6h, randomly selected 5 fields of the images, analyzed the results with Image J software, and the number of tubular structures was counted. For vasculogenic mimicry, the procedures as above, while the HepG2-NC and HepG2SG cells were used, and the tube formation was observed after 3–5 days.

### 2.11 Animal experiments

BALB/c nude mice (male, 5-weeks-old, weight 18~20 g) were purchased from the Guangzhou University of Chinese Medicine Laboratory Animal Center (Guangzhou, China). Tumor cells (5×10^6^ in 0.1 mL phosphate-buffered saline, PBS) were injected into the right axillary region randomly. For tumor formation, the mice were monitored two times per week for 4 weeks and then sacrificed by carbon dioxide asphyxiation. After the animal experiments, carcasses were returned to the Laboratory Animal Center for harmless treatment. The study was conducted in accordance with the ARRIVE guidelines (https://arriveguidelines.org). Animal experiments were approved by Rulge Biotechnology Committee for the Institutional Animal Care and Use (ethical approval number: 20230201002).

### 2.12 Hematoxylin and eosin (H&E) staining

The paraffin-embedded tissue sections were subjected to a series of washes for rehydration and clearing following dehydration. The sections were immersed in xylene twice, each for 20 minutes, followed by a graded ethanol series: processing twice with anhydrous ethanol (10 minutes each), 95% ethanol (5 minutes), 90% ethanol (5 minutes), 80% ethanol (5 minutes), and 70% ethanol (5 minutes). Subsequently, the sections were rinsed thoroughly with distilled water. For histological staining, the nuclei were stained using Harris hematoxylin solution for approximately 5 minutes. The sections were then differentiated with hydrochloric acid alcohol, blued in ammonia water, and rinsed again under running water. Cytoplasmic staining was performed by treating the sections with eosin solution for 1 to 3 minutes. After final dehydration steps, the sections were mounted with neutral balsam for microscopic examination.

### 2.13 Immunofluorescence

After dehydration, the sections were processed through xylene and gradient ethanol, then transferred to a retrieval box containing citrate antigen retrieval buffer, allowed to cool naturally, placed in PBS and washed three times. An autofluorescence quencher was applied to the sections for 5 min, and rinsed with running water for 10 min, added BSA in the circle and incubated for 30min, dropped the primary antibody (anti-CD206 antibody, 18704-1-AP, Proteintech, Rosemont, Illinois, USA; Rabbit anti-CD80 antibody, abs137159, Absin, Shanghai, China; Rabbit anti-SRGN antibody, bs-6789R, BIOSS, Beijing, China) on the slice. In a humid box, the slice was incubated at 4°C overnight, then added the secondary antibody in the circle to cover the tissue, and incubated for 50 min at room temperature in the dark. The slides were washed three times. DAPI staining solution was added to the circle and incubated for 10 min, and then the slides washed as above. After air-drying, the sections were added with anti-fluorescence quenching mounting medium and then examined under a fluorescence microscope, with images captured for further analysis.

### 2.14 Immunohistochemistry

Sections were sequentially deparaffinized and hydrated. For antigen retrieval, tissue sections, placed in a retrieval cassette filled with citrate-based antigen retrieval buffer, underwent heat-induced epitope retrieval. For endogenous peroxidase blocking, sections were incubated in 3% hydrogen peroxide solution at room temperature. After washing, 3% BSA was applied to for serum blocking. Then sections were incubated with PBS-diluted anti-CD34 antibody (YT0757, ImmunoWay, San Jose, CA, USA) overnight at 4°C in a humidified chamber. After washing, a horseradish peroxidase (HRP, BA1060, Bode)-conjugated secondary antibody was applied and incubated. Freshly prepared 3,3’-Diaminobenzidine (DAB, ab64238, Abcam) substrate solution was applied, and color was monitored under a microscope. For periodic acid-schiff (PAS, G1285, Solarbio, Beijing, China) staining, sections were treated with periodic acid solution, then incubated with PAS reagent. Nuclei were counterstained with hematoxylin. Stained sections were examined under a microscope, and images were acquired and analyzed. Microvessel density (MVD) was analyzed following the protocol of Weidner et al (46).

### 2.15 THP-1 cell culture and differentiation

THP-1 cells at a density of 5×10^5^ cells/mL per well were added in a six-well plate and cultured in RPMI1640 complete medium supplemented with 100 ng/mL of Phorbol 12-Myristate 13-Acetate (PMA, 16561-29-8, Solarbio) and 10% fetal bovine serum (FBS). After 48h of stimulation, the macrophages were successfully differentiated. M1 macrophages were induced by stimulation with 100 ng/mL LPS (L2880, Sigma-Aldrich, St. Louis, Missouri, USA) and 10 ng/mL IFN-γ (4Abiotech, Beijing, China) for 48h. For M2 macrophages, the cells were stimulated with 25 ng/mL IL-4(#200-04, PeproTech, Cranbury, NJ, USA) and 25 ng/mL IL-13(P5178, Beyotime, Haimen District, Jiangsu, China).

### 2.16 Co-culture of macrophages and hepatoma cells

M0 cells in the logarithmic growth phase were seeded into the lower chamber (0.3 μm) of the Transwell. HepG2-NC and HepG2SG cells were cultured in the upper chamber, respectively, maintaining the same serum concentration in both chambers. After 48 h, cells collected from the lower chamber were designated as tumor-associated macrophages TAM1 (co-cultured with HepG2-NC) and TAM2 (co-cultured with HepG2SG).

### 2.17 CCK8 assay for viability of hepatoma sells co-cultured with TAM1 and TAM2

TAM1 and TAM2 (in the upper chamber) were co-cultured in triplicates with HepG2 and Huh7 hepatoma cells (in the lower chamber) for 48h. Subsequently, HepG2 and Huh7 cells were seeded in a 96-well plate at a concentration of 1×10^4^ cells/100 μL per well. Other procedures were followed as step 2.8.

### 2.18 Transwell migration and invasion assays

Hepatoma cells at a density of 1×10^5^ were seeded in the upper chamber, added 100 μL serum-free DMEM. TAM1 or TAM2 with 600 μL DMEM were added to the lower chamber, followed by incubation at 37°C and 5% CO2 for 12h. After the chamber was removed, the cells on the upper chamber were carefully wiped away. The cells on the membrane were fixed with 4% paraformaldehyde for 20 min, followed by staining with crystal violet for 10 min. Images of the stained cells were captured for statistical analysis.

For invasion assay, HepG2 and Huh7 cells added to the upper chamber were co-cultured with TAM1 and TAM2 in the lower chamber, respectively, then other steps as step 2.9.

### 2.19 ELISA

Standards and samples were added 100 μL per well to the corresponding wells, with sample diluent serving as blank. After 48 h, the supernatant was collected and ELISA was performed according to the manufacturer’s instructions. Finally, 50 μL stop solution was added to each well, the results of SRGN, arginase1(Arg1), inducible nitric oxide synthase(iNOS2), interleukin 1β(IL-1β), vascular endothelial growth factor A (VEGF-A) and matrix metalloproteinase-9 (MMP9) were read at the absorbance of 450 nm, with 620 nm as the calibration wavelength. ELISA kits were purchased from Elabscience (Wuhan, China).

### 2.20 Flow cytometry

For surface marker staining, M2 macrophages, TAM1, and TAM2 cells were incubated with fluorescently-labeled antibodies against CD80 (FITC-conjugated, clone 2D10.4, 11-0809-42, eBioscience™, San Diego, CA, USA) and CD206 (APC-conjugated, clone 19.2, 17-2069-42, eBioscience™) at room temperature for 20 minutes in the dark. After washing with PBS by centrifugation at 1000rpm for 10min, the cells were fixed using 100 μL of Fix&Perm Reagent A for 15 minutes. Finally, the stained cells were resuspended in 0.2 mL PBS for immediate flow cytometry analysis.

### 2.21 Statistical analysis

Measured data were compared using t-tests or ANOVA using SPSS 22.0 (IBM Corp. Armonk, NY, USA), and the counted data were compared using χ^2^ tests. Differences between means for data with skewed distribution and variance were analyzed using rank-sum tests. Statistical significance was set at P < 0.05.

## 3 Results

### 3.1 Single-cell transcriptomics reveals SRGN expression heterogeneity in HCC

**Figure 1:**
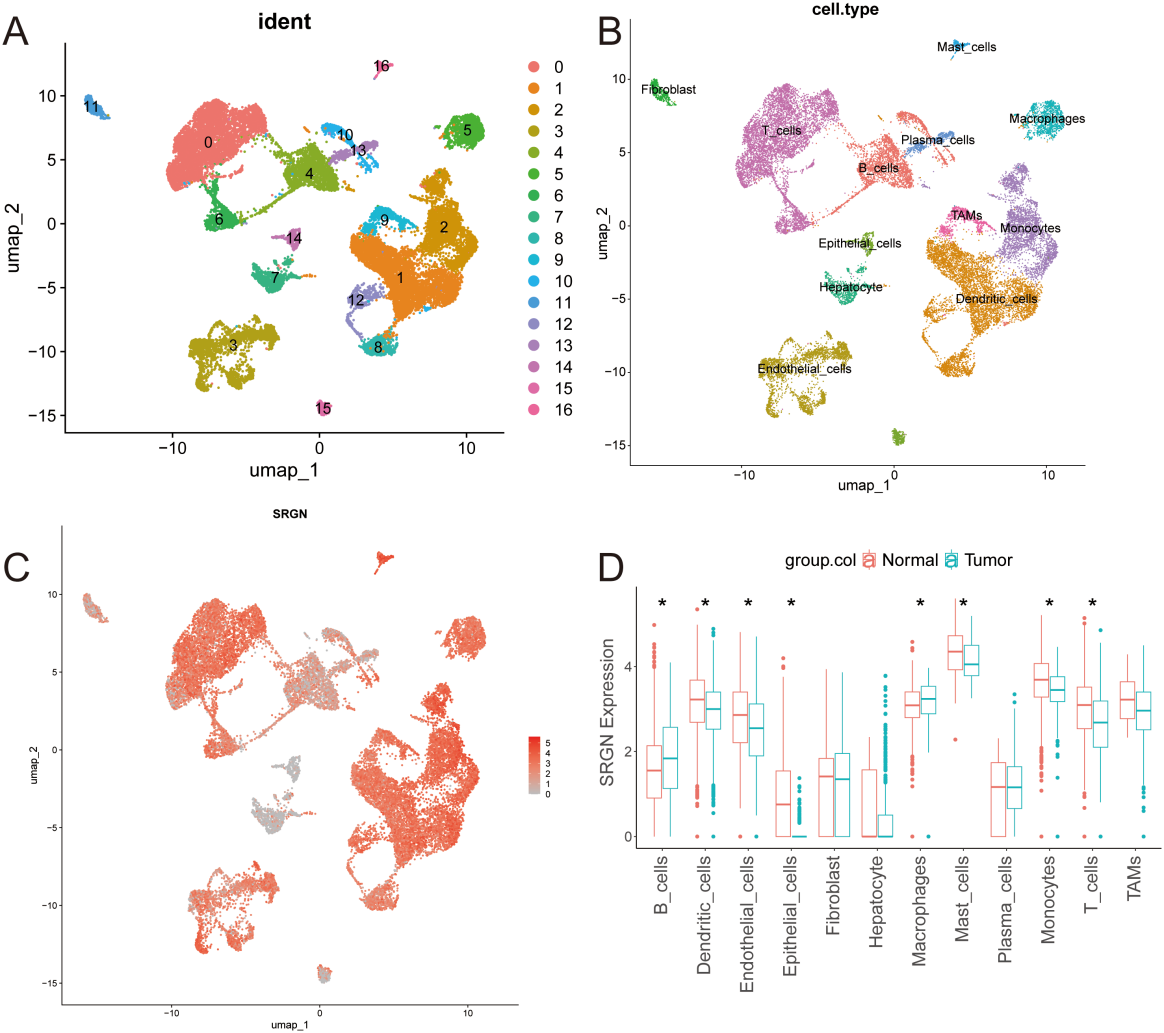
Single-cell analysis of SRGN expression in HCC. **(A)** The UMAP plot of all cells after quality control and standardization revealed 17 cell clusters marked with different colors. **(B)** The 17 clusters were annotated as 12 types of cells. **(C)** Feature plots showed SRGN expression across different cell types. **(D)** Comparative analysis of SRGN expression between HCC and normal group was presented in box plot. “*” denotes a p-value < 0.05.

Single-cell sequencing data of HCC and corresponding normal tissue samples from five patients were retrieved from the GEO database. After rigorous quality control, dimensionality reduction, and clustering analysis, 17 distinct cell subpopulations were identified (**Figure 1A**). Further cell annotation, along with umap visualization, classified these cells into 12 types: B cells, dendritic cells, endothelial cells, epithelial cells, fibroblast, hepatocyte, macrophages, mast cells, plasma cells, monocytes, T cells, and tumor-associated macrophages (TAMs) (**Figure 1B**). SRGN gene expression was nearly ubiquitous across all cells, being especially prominent in dendritic cells, endothelial cells, macrophages, fibroblast, mast cells, plasma cells, monocytes, T cells, and TAMs (**Figure 1C**). Comparative analysis of normal and tumor tissues revealed significantly higher SRGN expression in B cells and macrophages of tumor samples than in normal tissue cells (**Figure 1D**). Subsequently, we utilized the CROST database (47) to analyze SRGN’s spatial localization and gene expression in different HCC tissue samples. The cell types of VISDS000514, VISDS000454, VISDS000507, and VISDS000513 are shown in **Supplementary Figures S1A, C, E, G**, respectively. Our findings demonstrated high SRGN expression in HCC tissues, particularly in macrophages of VISDS000514 (**Supplementary Figure S1B**), VISDS000454 (**Supplementary Figure S1D**), VISDS000507 (**Supplementary Figure S1F**), and VISDS000513 (**Supplementary Figure S1H**).

### 3.2 SRGN expression and prognosis in associated with TME of LIHC

**Figure 2:**
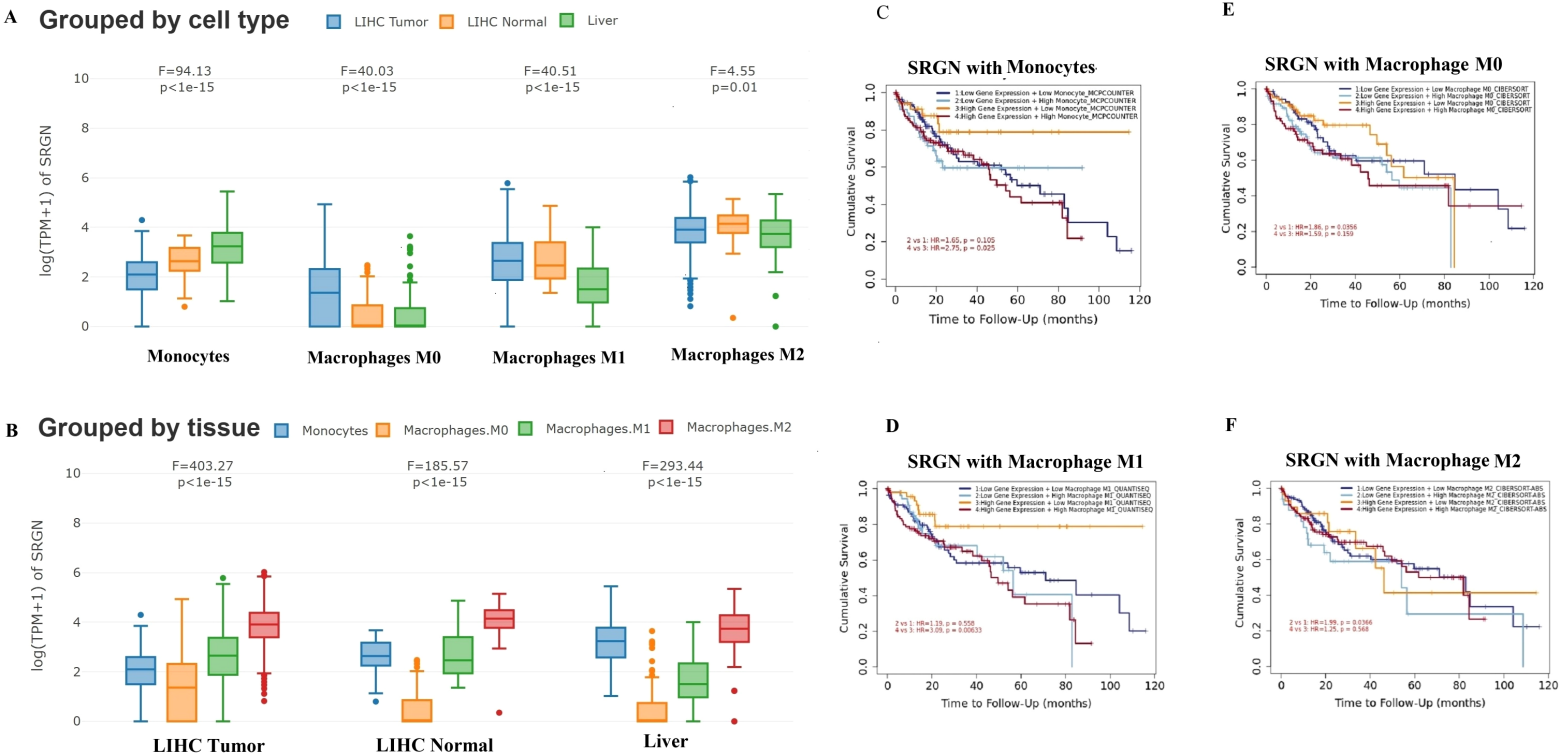
SRGN expression and prognosis in monocyte/macrophage subsets in LIHC. GEPIA analysis using ANOVA test using TCGA/GTEx sub-datasets: **(A)** SRGN expression in LIHC tumor, LIHC normal and liver tissues grouped by cell type. **(B)** SRGN expression in monocyte/macrophage subsets grouped by tissue of LIHC tumor, LIHC normal and liver, respectively. TIMER analysis using the Cox proportional hazards model: **(C–F)** SRGN prognosis with monocyte, macrophageM1, M0 and M2 in LIHC.

Building on the observation of SRGN’s prominent expression in macrophages within the TME of HCC, we further explored the relationship between SRGN expression and specific macrophage subsets, along with its prognostic implications in LIHC. **Figure 2** provides a more in-depth analysis of SRGN expression across different macrophage subsets in LIHC. GEPIA analysis revealed that in LIHC tumor tissues, SRGN expression was highest in M2 macrophages, followed by M0 and M1 macrophages, when compared to liver tissues and LIHC-adjacent normal tissues (**Figures 2A, B**). This pattern highlights SRGN’s potential role in modulating macrophage polarization within the TME, potentially promoting a pro-tumorigenic M2 phenotype.

To determine whether macrophage-intrinsic SRGN is functionally relevant, we re-annotated macrophages from the single-cell data into SRGN_High and SRGN_Low subsets (median split; **Supplementary Figure S6A**). SRGN_High macrophages exhibited a striking up-regulation of pro-angiogenic pathways (VEGF, EGFR, MTORC1 and VEGFR2-mediated permeability) in GSEA (**Supplementary Figures S6B, C**), suggesting that SRGN expression within macrophages themselves may also contribute to neovascularisation.

Differential expression analysis across pan-cancer via TIMER showed that SRGN RNA levels in tumor tissues were significantly lower than those in adjacent normal tissues in LIHC, breast invasive carcinoma (BRCA), lung squamous cell carcinoma, and LUAD (**Supplementary Figure 1**). However, GEPIA analysis of RNA-Seq data showed no significant difference in SRGN expression between LIHC and normal tissues, and no significant prognostic association in LIHC (**Supplementary Figure 2**; **Supplementary Table 1**). TIMER analysis suggested that SRGN expression was closely related to TME cells (**Supplementary Tables 2**, **3**), which should be considered in multivariate Cox regression models. Notably, Kaplan–Meier plotter multivariate analysis, adjusted for clinical confounders such as stage, grade, AJCC_T stage, demographic factors, treatment history, and vascular invasion, suggested SRGN mRNA as a favorable prognostic indicator for overall survival (OS) and progression free survival (PFS), but an unfavorable one for disease-specific survival (DSS) and relapse-free survival (RFS) (**Supplementary Table 4**).

TIMER analysis using the Cox proportional hazards model further elucidated the relationship between SRGN expression and monocyte/macrophage subsets in LIHC (**Figures 2C–F**). The analysis indicated that SRGN expression was significantly associated with the infiltration of various immune cells, including macrophages. In the cohort with high SRGN expression, high infiltration of MDSCs, M1 macrophages, and monocyte_MCPCOUNTER was associated with unfavorable OS, whereas high infiltration of CD8+ T cells, resting memory CD4+ T cells, ECs, and HSCs was associated with favorable prognosis for OS. In the cohort with low SRGN expression, high infiltration of macrophage_TIMER, M0_CIBERSORT, and M2_CIBERSORT-ABS were unfavorable factors for OS. These findings suggest that SRGN may influence the tumor microenvironment by modulating the infiltration and activity of different immune cell subsets, particularly macrophages, thereby impacting patient prognosis.

### 3.3 Functional enrichment analysis of SRGN

**Figure 3:**
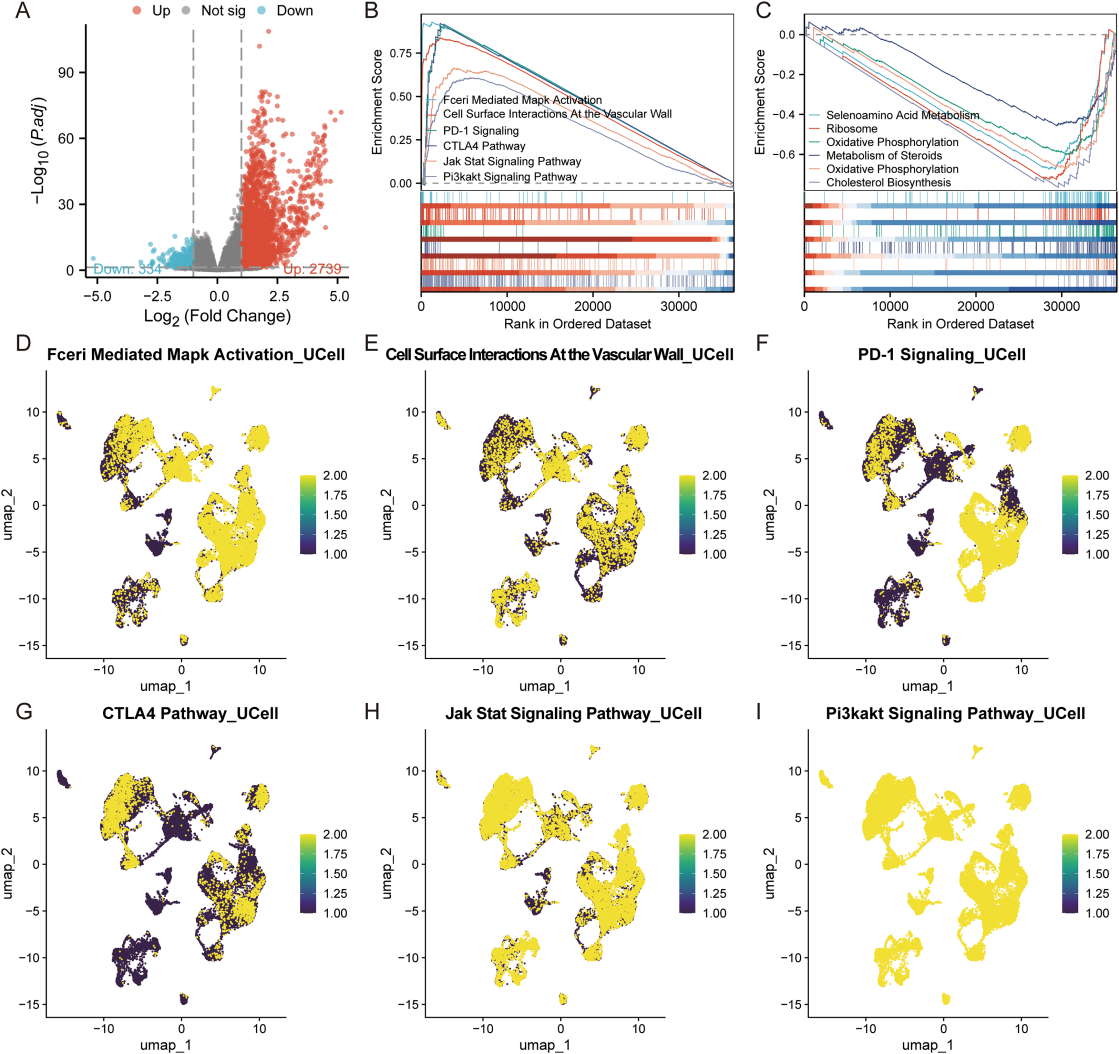
GSEA enrichment analysis for the SRGN. **(A)** Volcano plot for single gene differential analysis of SRGN. **(B)** Pathways upregulated in the high-SRGN group. **(C)** Pathways downregulated in the low-SRGN group. **(D–I)** Localization of different pathways in the single-cell dataset.

We stratified the TCGA-LIHC cohort into high-risk and low-risk categories based on the median expression of SRGN. Following this stratification, we performed a differential expression analysis, comparing the high-risk and low-risk groups using thresholds of logFC > 1 or < -1 and p < 0.05. The results of this analysis are illustrated in the volcano plot shown in **Figure 3A**. Subsequently, we conducted GSEA on the differentially expressed genes, revealing six upregulated pathways (**Figure 3B**) and six downregulated pathways (**Figure 3C**). The upregulated pathways included FCERI-mediated MAPK activation, cell surface interactions at the vascular wall, PD-1 signaling, the CTLA4 pathway, the JAK-STAT signaling pathway, and the PI3K-AKT signaling pathway. Conversely, the downregulated pathways encompassed selenoamino acid metabolism, ribosome biogenesis, oxidative phosphorylation, steroid metabolism, and cholesterol biosynthesis.

In addition, we performed enrichment analysis on the six upregulated pathways within the single-cell dataset of HCC. Our findings indicate that FCERI-mediated MAPK activation is upregulated across various cell types, including TAMs, macrophages, plasma cells, mast cells, B cells, dendritic cells, and monocytes. The pathway of cell surface interactions at the vascular wall showed upregulation in macrophages, plasma cells, B cells, endothelial cells, and monocytes. PD-1 signaling was found to be elevated in TAMs, macrophages, dendritic cells, T cells, and monocytes. The CTLA4 pathway exhibited increased expression in TAMs, macrophages, dendritic cells, T cells, and monocytes. Likewise, the JAK-STAT signaling pathway was upregulated in macrophages, dendritic cells, endothelial cells, T cells, and monocytes, while the PI3K-AKT signaling pathway showed upregulation specifically in fibroblasts and endothelial cells. Notably, macrophages were involved in nearly all of the aforementioned pathways (**Figures 3D–I**).

### 3.4 The immune infiltration analysis of SRGN

**Figure 4:**
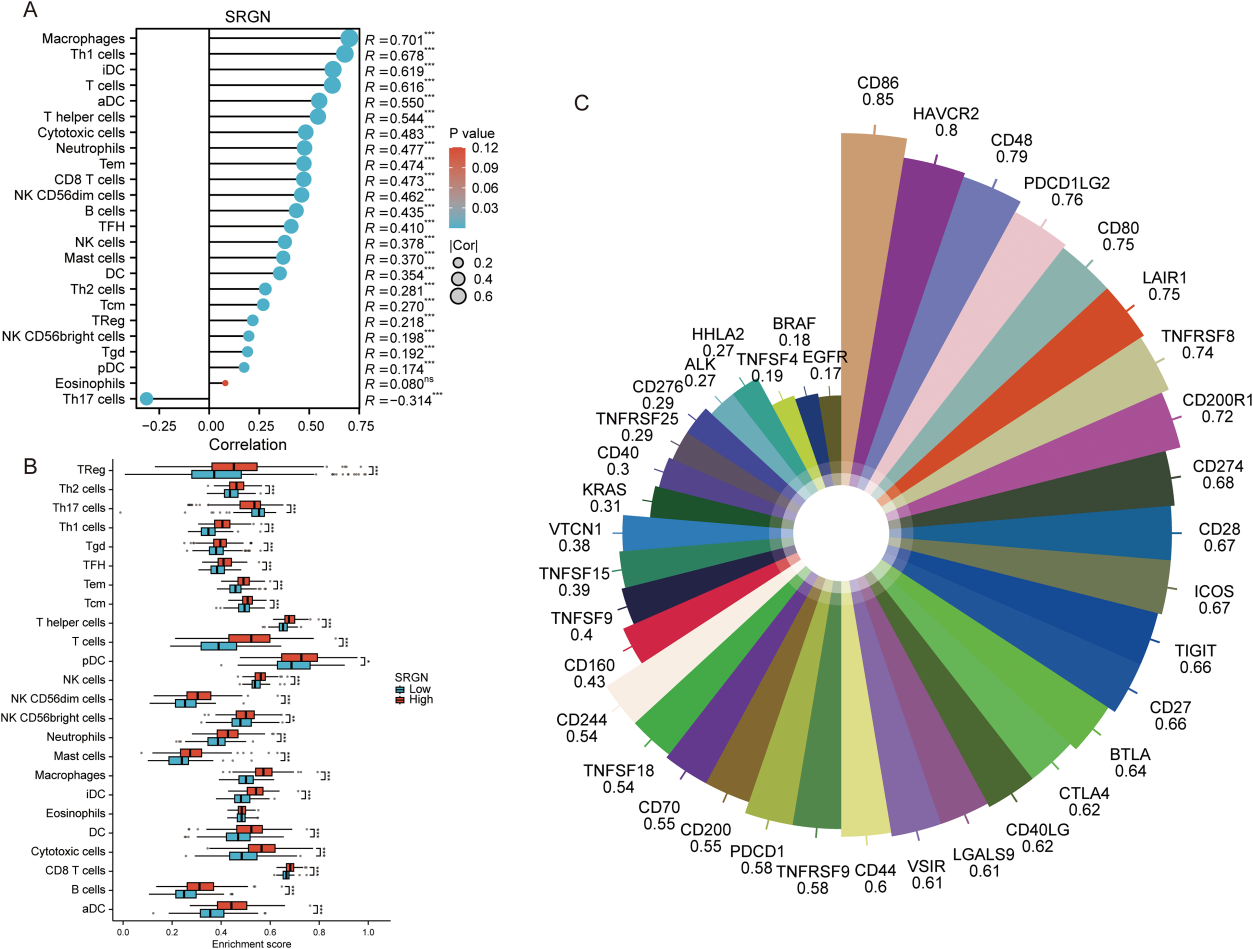
The immune infiltration analysis of SRGN. **(A)** Correlation analysis results between SRGN and immune infiltrating cells. **(B)** Comparison of ssGSEA immune infiltrating cell proportions between high- and low-risk groups based on SRGN expression. **(C)** Correlation analysis of SRGN expression with immune checkpoint genes.

We subsequently analyzed the infiltration of 24 immune cell types in hepatocellular carcinoma tissues using single-sample Gene Set Enrichment Analysis (ssGSEA). The correlation between the expression levels of SRGN in TPM format and the levels of immune cell infiltration was assessed using Spearman correlation tests. The results revealed a negative correlation between SRGN expression and Th17 cells, while a positive correlation with the majority of immune cell types was observed. Notably, the highest correlation was found with macrophages, yielding a correlation coefficient of 0.7 (**Figure 4A**). Furthermore, stratifying by the median expression of SRGN, we discovered that the high SRGN expression group exhibited significantly higher infiltration scores for most immune cells, including macrophages, compared to the low SRGN expression group (**Figure 4B**). Given that the GSEA analysis identified PD-1 signaling and the CTLA4 pathway as upregulated in the high SRGN expression cohort, we proceeded to analyze the correlation between SRGN and immune checkpoint genes. Our findings indicated a positive correlation between SRGN and 40 immune checkpoint genes. The specific immune checkpoint genes and their respective correlation coefficients are detailed in **Figure 4C**. Notably, while the P-value for EGFR was 0.001, the P-values for all other immune checkpoint genes were less than 0.001. This indicates that high expression of SRGN is closely associated with an immune activation status in hepatocellular carcinoma.

### 3.5 Cell cycling stage analysis of SRGN

**Figure 5:**
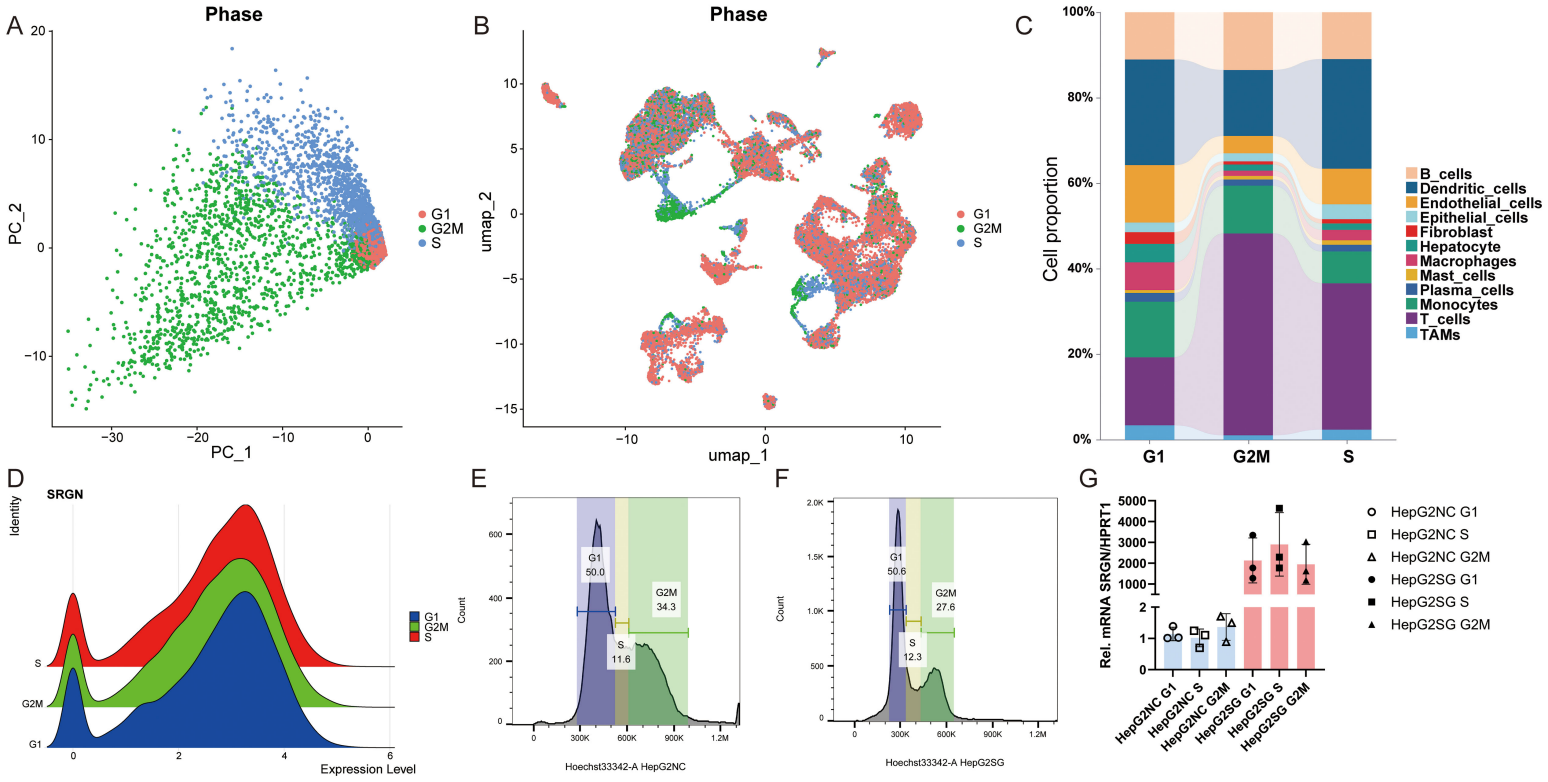
Cell cycling stage analysis. **(A)** PCA plots illustrate the cell cycle phases of the single-cell dataset. **(B)** UMAP of single-cell transcriptomes colored by cell cycle phase (G1/G2M/S) via Seurat’s CellCycleScoring. **(C)** Cell cycle distribution of different cells. **(D)** Cell cycling stage analysis of SRGN. HepG2-NC **(E)** and HepG2SG **(F)** cells were stained with Hoechst-33342, sorted into G1/S/G2/M populations, and subjected to qPCR **(G)**. Axis ranges differ due to staining intensity but gates were set per sample based on DNA content.

Cell cycle activity was quantified using the CellCycleScoring function in Seurat, which computed S-phase and G2/M-phase gene set scores to classify cells into distinct cycle phases. PCA revealed clear separation along PC1, with S-phase cells clustering in the upper quadrant and G2/M-phase cells in the lower quadrant (**Figure 5A**), while UMAP demonstrated partial segregation of these populations (**Figure 5B**). Cell-type–specific analysis showed heterogeneous distributions: macrophages, endothelial cells, and dendritic cells were enriched in G1/S phases, whereas B cells and hepatocytes were predominantly G2/M-phase–enriched; T cells exhibited increased G2/M and S-phase proportions, and TAMs were enriched in G1/S phases (**Figure 5C**). Critically, SRGN exhibited consistently high expression across all three phases (G1, S, and G2/M), indicating its potential role in cell cycle regulation independent of phase-specific transcriptional programs. These findings collectively suggest SRGN as a cell cycle–associated factor in the HCC microenvironment. To experimentally verify the single-cell observation that SRGN transcript levels remain constant in G1, S and G2/M, we flow-sorted HepG2-NC and HepG2SG cells after Hoechst-33342 staining (**Figures 5E, F**). qPCR of three independent sorts showed that SRGN mRNA was barely detectable and essentially unchanged across the cycle phases in NC cells, whereas it was markedly elevated in HepG2SG cells without significant cycle-dependent fluctuation (**Figure 5G**). Thus, SRGN abundance remained uniformly high across G1, S and G2/M phases, mirroring the cycle-independent pattern observed in single-cell data.

### 3.6 Pseudotime trajectory and cellular communication in the HCC microenvironment

Based on the relationship between SRGN and macrophages, we initiated our analysis from macrophages and employed Monocle3 for single-cell trajectory analysis (**Supplementary Figure S7A**). Subsequently, we conducted a bulk differential gene analysis of time-series macrophage genes. The heatmap demonstrated six clusters of differentially expressed genes (**Supplementary Figure S7B**). The trend line diagram indicated that the expression dynamics of SRGN gradually increased over time, with macrophages from the normal group exhibiting higher expression levels during the early developmental stages, while those from the tumor group showed enhanced expression in the later developmental stages (**Supplementary Figure S7C**).

**Figure 6:**
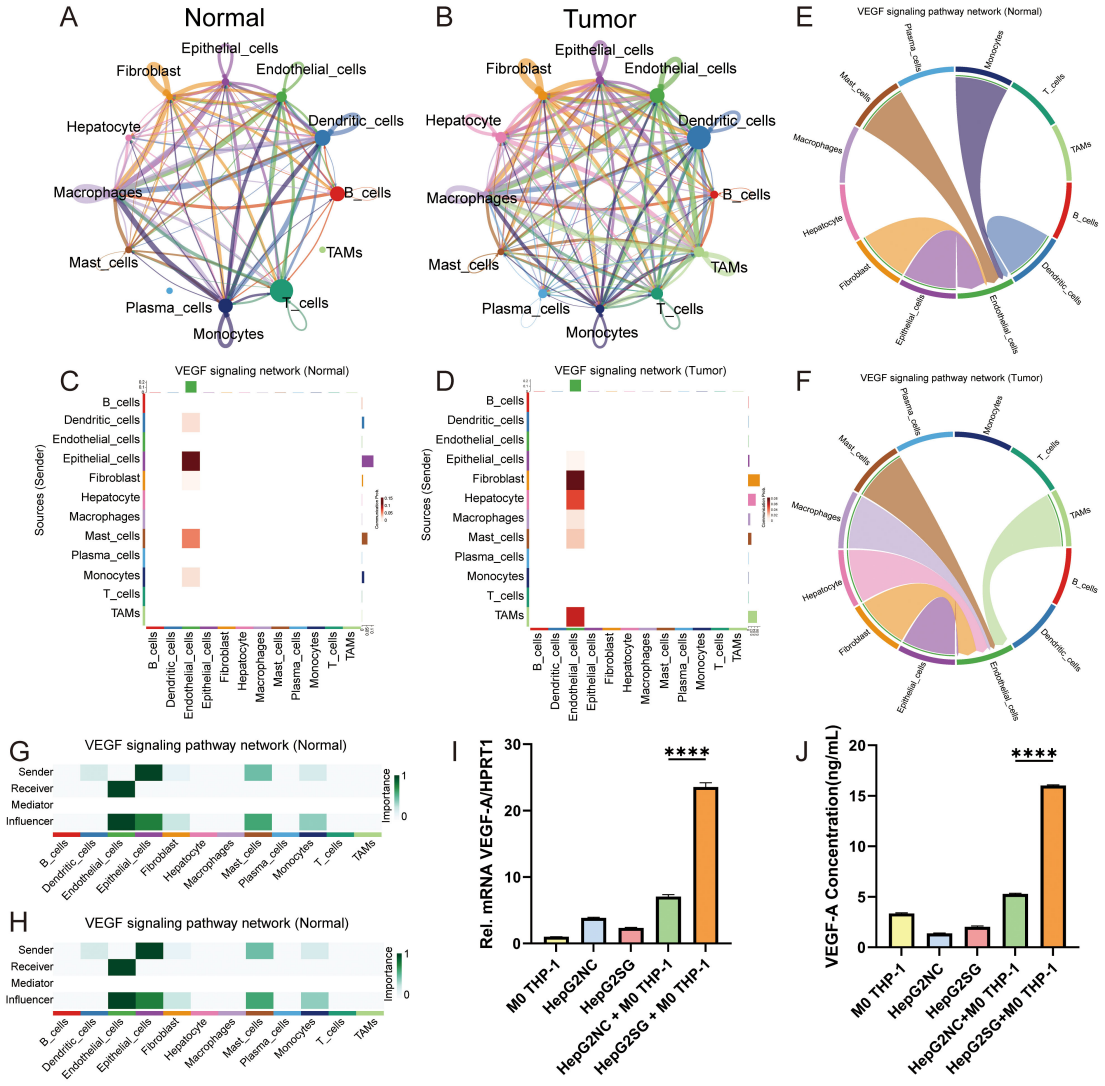
Cell–cell communication analysis in HCC. **(A)** Circle diagram showing the number of cell communication interactions among different groups cell in the Normal group. **(B)** Circle diagram showing the number of cell communication interactions among different cell groups in the Tumor group. Cell-type to cell-type heatmap of VEGF signaling communication probabilities in normal **(C)** and tumor **(D)** groups. Difference and chord diagram of the VEGF signaling pathway between normal **(E)** and tumor **(F)** groups. Heatmaps of the VEGF signaling pathway network displaying the participation of each cell type in cell communication in normal **(G)** and tumor **(H)** groups. **(I, J)** Experimental validation of VEGF-A signaling. **(I)** VEGFA mRNA levels in lower-chamber M0 THP-1 macrophages after co-culture (****P < 0.001). **(J)** VEGF-A protein levels in 48-h co-culture supernatants measured by ELISA (****P < 0.001).

To explore potential intercellular interactions, we utilized CellChat to assess cell communication within the hepatocellular carcinoma dataset GSE242889. The results indicated that overall signaling activity was significantly lower in the normal group compared to the tumor group (**Figures 6A, B**). Analysis of regulatory relationships revealed that within the tumor microenvironment, macrophages exhibited heightened interactions with T cells, tumor-associated macrophages (TAMs), and endothelial cells. Plasma cells in the tumor group also showed increased communication with other cell types. Given that Gene Set Enrichment Analysis (GSEA) identified enrichment for cell surface interactions at the vascular wall, we focused on the VEGF signaling network from a cell communication perspective. VEGF comprises a family of signaling proteins that promote angiogenesis. In the normal group, communication probability among macrophages, TAMs, and endothelial cells was negligible, while fibroblasts displayed minimal involvement (**Figure 6C**). In contrast, the tumor group demonstrated enhanced communication probabilities between macrophages, TAMs, fibroblasts, and endothelial cells, particularly targeting endothelial cells (**Figure 6D**). We visualized the VEGF signaling pathway using chord diagrams for both the normal (**Figure 6E**) and tumor (**Figure 6F**) groups. Further dissection of the VEGF network revealed that macrophages in the normal group did not contribute significantly (**Figure 6G**), whereas in the tumor group, macrophages displayed high expression levels and elevated importance as both Sender and Influencer nodes (**Figure 6H**).

To functionally validate the enhanced VEGF signaling network predicted by CellChat, we conducted co-culture experiments using HepG2 cells and M0-THP-1 macrophages. We first measured VEGFA mRNA levels in macrophages recovered from the co-culture system. VEGFA transcript levels were more than threefold higher under SRGN-overexpressing HepG2 (HepG2SG) conditions compared to control (HepG2-NC) co-cultures (**Figure 6I**). Consistent with the mRNA data, ELISA analysis of 48-hour supernatants confirmed a significant increase in VEGF-A protein secretion in SRGN-OE-HepG2 + M0-THP-1 co-cultures relative to control (**Figure 6J**). Collectively, these data demonstrate that SRGN-high hepatoma cells actively drive VEGF-A production, particularly in macrophages, thereby confirming the computationally predicted upregulation of VEGF-mediated intercellular communication in the tumor microenvironment.

### 3.7 *In vitro* and *in vivo* pro-tumorigenicity of SRGN

**Figure 7:**
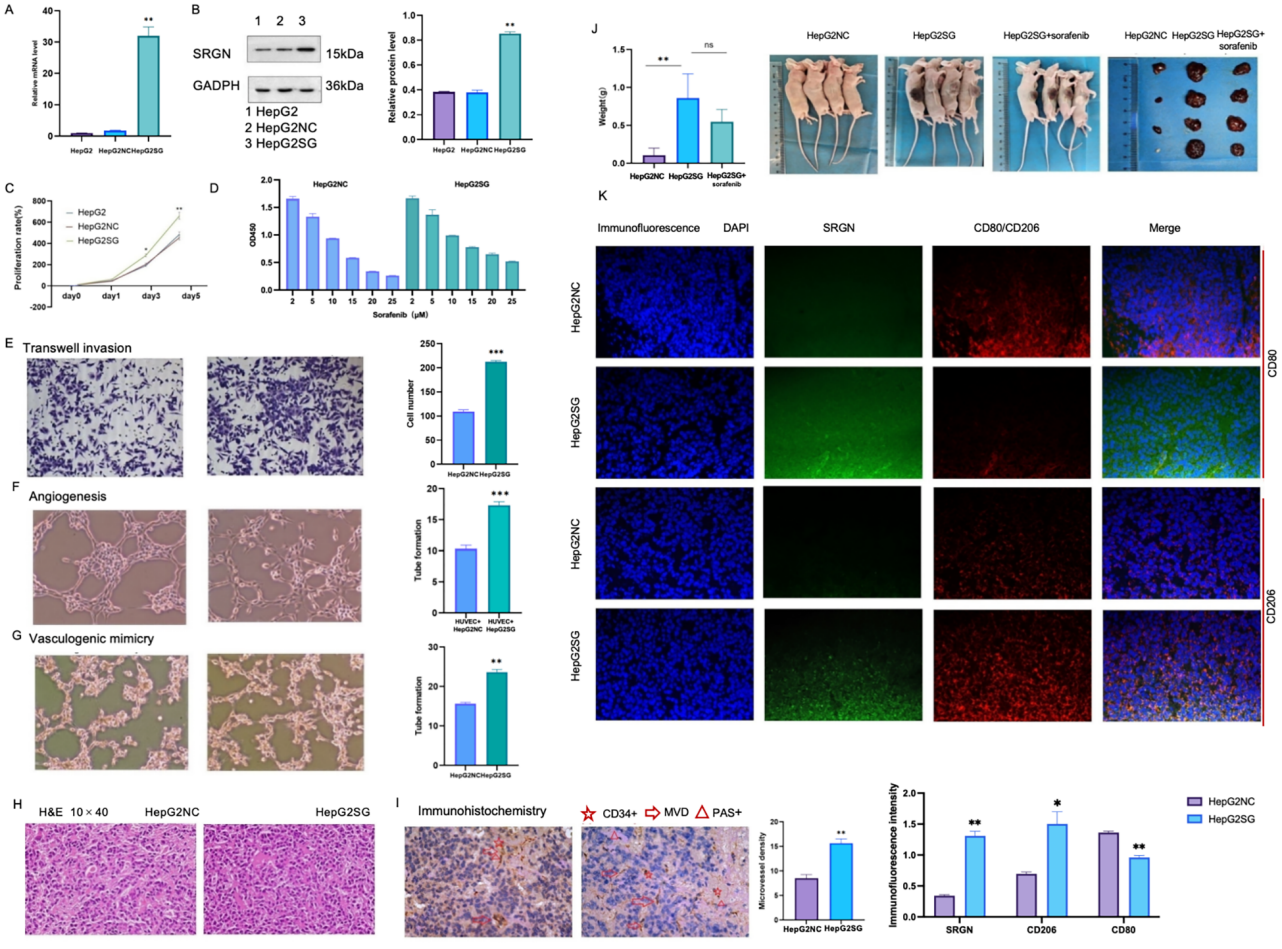
*In vitro* and *in vivo* experiments of pro-tumorigenicity of SRGN. **(A)** Relative expression of SRGN in HepG2, HepG2SG (overexpressing SRGN) and HepG2-NC (transfected with null vector control) examined by quantitative reverse transcription-polymerase chain reaction. **(B)** Expression of SRGN protein in HepG2, HepG2SG and HepG2-NC cells examined by western blot. **(C)** Proliferation rates (%) of HepG2, HepG2SG and HepG2-NC cells using CCK8 assay (^*^p < 0.05, ^**^p < 0.01). **(D)** Proliferation rates (%) of HepG2SG and HepG2-NC cells treated with sorafenib, resistance index=1.15. **(E)** Invasion of HepG2SG and HepG2-NC cells examined by transwell assay (^***^p < 0.001). **(F)** Angiogenesis of HUVECs cultured with supernatant from HepG2SG and HepG2-NC cells, respectively (^***^p< 0.001). **(G)** Vasculogenic mimicry of HepG2SG and HepG2-NC cells (^**^p< 0.01). **(H)** Representative hematoxylin and eosin staining of tumor sections from mice xenograft with SRGN overexpressing HepG2SG and control cells. **(I)** Representative immunohistochemistry for CD34 and PAS staining and microvessel density. **(J)** Weight of HepG2SG and HepG2-NC subcutaneous xenograft tumors in nude mice (^**^p < 0.01). **(K)** Immunofluorescence staining of subcutaneous tumor tissues. Both SRGN and CD206 were up-regulated while CD80 was down-regulated in HepG2SG mice compared with HepG2-NC mice (^*^p < 0.05, ^**^p < 0.01). Light blue: DAPI; green: fluorescein isothiocyanate-stained SRGN; red: CY3-stained CD80 or CD206.

SRGN promoted the proliferation of SRGN-overexpressing HepG2SG cells compared to HepG2-NC cells (transfected with blank control vector) and HepG2 cells (**Figures 7A–C**, ^*^p< 0.05, ^**^p < 0.01). HepG2SG cells showed sorafenib resistance (resistance index 1.15, **Figure 7D**). HepG2SG cells also showed increased cell invasion compared to HepG2-NC cells in the transwell assay (**Figure 7E**, ^***^p< 0.001). The number of tubular structures was higher in human umbilical vein ECs (HUVECs) cultured with HepG2SG supernatant than in those cultured with HepG2-NC supernatant (**Figure 7F**, ^***^p< 0.001). SRGN promoted vascular mimicry tube formation in HepG2SG cells compared to that in HepG2-NC cells (**Figure 7G**, ^**^p< 0.01). Hematoxylin and eosin staining showed that the cells were distributed in clumps or strands in subcutaneous tumor tissues (**Figure 7H**). IHC staining for CD34 and PAS staining demonstrated enhanced tumor angiogenesis, as evidenced by a higher microvessel density in the HepG2SG group compared to the HepG2-NC group (**Figure 7I**, ^**^p< 0.01). Subcutaneous xenograft tumors derived from HepG2SG cells were heavier than those derived from HepG2-NC cells (**Figure 7J**, ^**^p< 0.01). SRGN and CD206 were upregulated, whereas CD80 was downregulated in subcutaneous tumor tissues in HepG2SG xenograft mice compared with HepG2-NC mice (**Figure 7K**, ^*^p < 0.05, ^**^p < 0.01).

**Figure 8:**
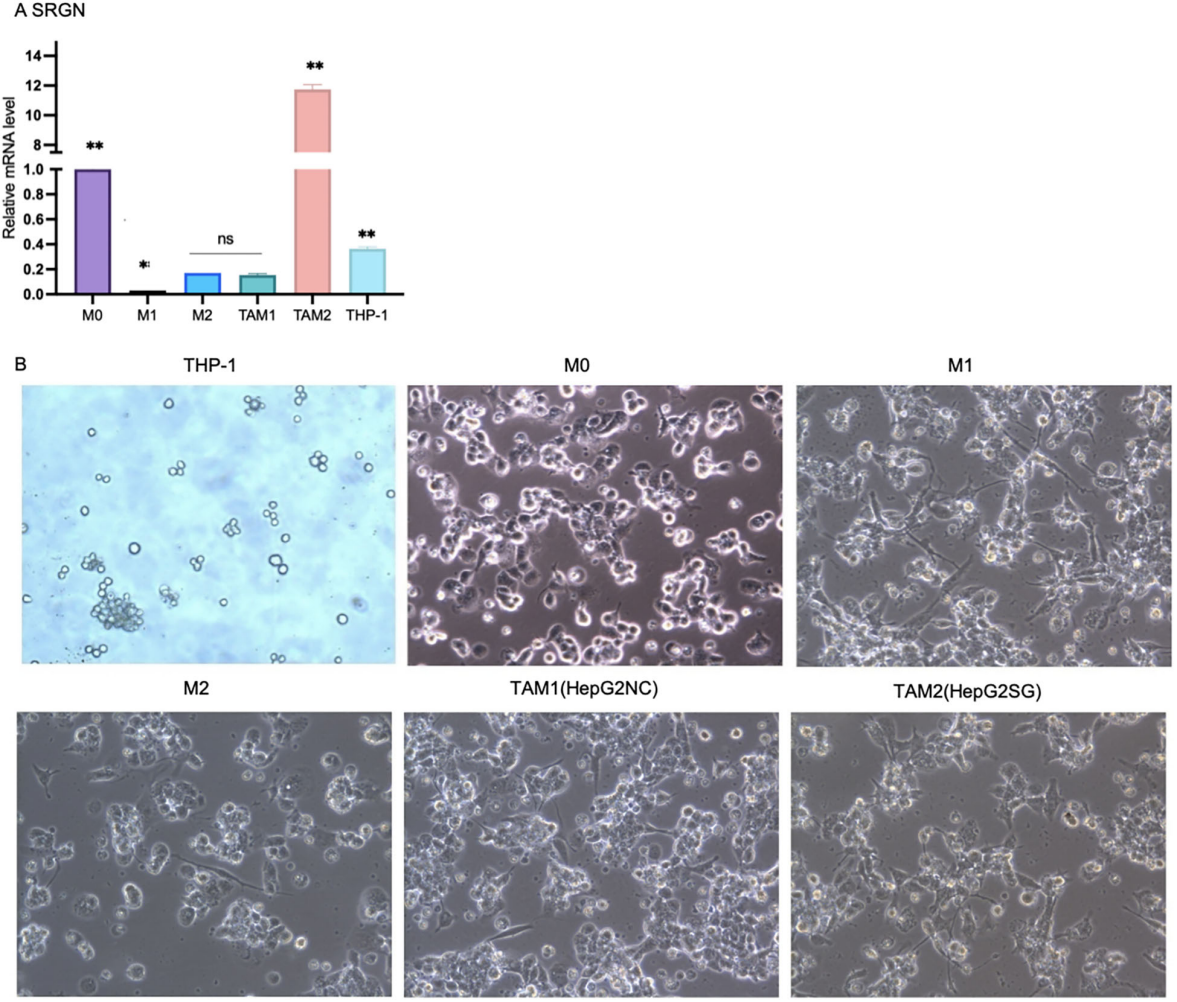
The SRGN mRNA expression in THP-1 cells, M0 macrophages, M1 and M2 macrophages, as well as TAM1 and TAM2. SRGN mRNA levels were significantly different among monocyte/macrophages (**p < 0.01). Tumor -associated macrophages 1(TAM1): M0 co-cultured with HepG2-NC, TAM2: M0 co-cultured with SRGN overexpressing HepG2SG. NS: no significant difference.

The SRGN mRNA expression was examined in THP-1 cells, M0 macrophages, M1 and M2 macrophages, as well as tumor-associated macrophages (TAMs) differentiated by HepG2SG or HepG2-NC cells. Through pairwise comparison, SRGN mRNA levels were found to be significantly different (**Figure 8A**, ^**^p < 0.01), except between TAM1 and M2 (**Figure 8A**, NS: no significant difference). Among all the cell types, SRGN levels were highest in TAM2, with M0 macrophages having the second-highest increase, yet it was ten times lower than that in TAM2, but higher than THP-1 monocytes. This indicates that SRGN levels are regulated according to the differentiation/polarization of monocytes/macrophages, and SRGN-overexpressing HepG2 may enhance SRGN transcription in macrophages through a positive-feedback loop. Under the microscope, M0 macrophages derived from THP-1 cells induced by PMA exhibited an adherent and irregular shape. These M0 macrophages polarized into M1 and M2 macrophages. M1 macrophages displayed pseudopods and distinct bifurcations, while M2 macrophages exhibited a more uniform morphology, lacking apparent bifurcations. Tumor-associated macrophages 1 (TAM1, co-cultured with HepG2) and TAM2 (co-cultured with SRGN- overexpressing HepG2) demonstrated morphologies characteristic of both M1 and M2 macrophages (**Figure 8B**).

**Figure 9:**
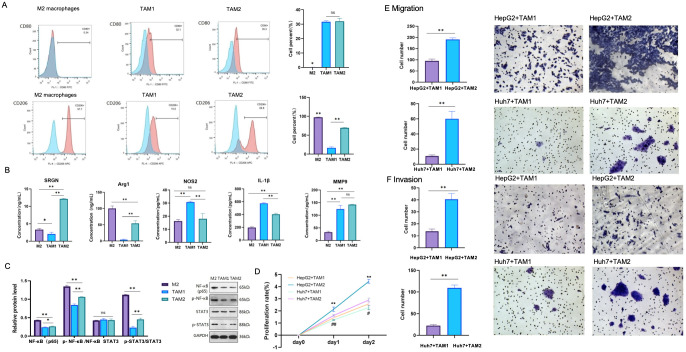
**(A)** CD80 and CD206 examined by flow cytometry in M2 macrophages, TAM1 and TAM2 (**p < 0.01, *p < 0.05). **(B)** SRGN, Arg1, NOS2, IL-1β and MMP9 were examined by ELISA in supernatants of M2 macrophages, TAM1 and TAM2 (**p<0.01, *p < 0.05); **(C)** NF-κB, Stat3 and phosphorylation in M2 macrophages, TAM1 and TAM2 (**p<0.01, *p<0.05). NS: no significant difference. **(D)** Cell proliferation of HepG2 and Huh7 cells induced by TAM1 and TAM2 respectively (**, ##p<0.01, # p<0.05). **(E)** Transwell migration of HepG2 and Huh7 cells induced by TAM1 and TAM2 respectively (**, ##p<0.01). **(F)** Transwell invasion of HepG2 and Huh7 cells induced by TAM1 and TAM2 respectively (**, ##p<0.01).

By flow cytometry, CD206 expression was significantly higher in TAM2 than in TAM1, but lower than in M2 macrophages. The level of CD80 showed no significant difference between TAM2 and TAM1, although CD80 expression was hardly indicated in M2(**Figure 9A**, ^*^p < 0.05, ^**^ p < 0.01). On day 1 and 2, the proliferation rate of HepG2 and Huh7 cells co-cultured with TAM2 cells was significantly higher than that with TAM1 cells (**Figure 9D**, ^**,##^p ≤ 0.01, ^#^ p<0.05). The number of migration and invasion HepG2 and Huh7 cells co-cultured with TAM2 cells was significantly greater than that with TAM1 cells, respectively (**Figures 9E, F**, ^**,##^p<0.01).

The levels of Arg1 and SRGN in TAM2 were significantly higher than that in TAM1. Conversely, iNOS2 and IL-1β levels were significantly lower in TAM2 compared to TAM1. However, MMP9 levels showed no significantly difference between TAM1 and TAM2 (**Figure 9B**, ^**^p <0.01, ^*^p < 0.05). NF-κB and phosphorylation p65, phosphorylation Stat3 were mostly activated in M2 macrophages, which were secondly increased in TAM2 (**Figure 9C**, ^**^p <0.01, ^*^ p <0.05). This suggested that NF-κB and STAT3 signaling pathways might be involved in SRGN-promoted TAMs.

## 4 Discussion

Using bioinformatics analyses, we comprehensively investigated the expression and prognostic significance of SRGN in PLC, particularly its crosstalk with macrophages. The pro-tumorigenicity of SRGN was further validated *in vitro* and *in vivo* experiment.

The role of SRGN in LIHC exhibits marked controversy across bioinformatics platforms. While TIMER data indicate reduced SRGN mRNA levels in LIHC versus adjacent tissues, Oncomine reports opposing elevation (48), with GEPIA showing no significance and KM-plotter revealing platform-dependent discrepancies (GeneChip vs. RNA-seq). Comprehensive prognostic analysis of SRGN in LIHC demonstrates context-specific heterogeneity in its clinical associations. Bioinformatics analyses (GEPIA/TIMER 2.0) revealed no significant prognostic association between SRGN mRNA levels and LIHC outcomes when TME cells were excluded. Notably, KM-plotter multivariate analysis adjusted for clinicopathological confounders (stage, grade, AJCC_T stage, demographic factors, sorafenib treatment, and vascular invasion) identified SRGN mRNA as a favorable prognostic indicator, whereas its protein expression predicted poorer overall survival and increased recurrence risk (10, 28). Similar discrepancy occurred between the present study about other cancers and several published reports (20, 21, 48, 49). In both breast and lung cancer tissues, SRGN was downregulated compared to normal adjacent tissues and correlated with inconsistent clinical outcomes (**Supplementary Table 1**; **Figures 1A, B**).

Menyhárt et al. reviewed 318 genes related to HCC survival and showed that none had an equivalent prognostic value to that of tumor stage. They also noted that the survival results according to protein expression were inconsistent with those of the transcriptome (50). The prognostic capability of SRGN may also vary depending on the covariates in the Cox regression such as clinicopathological factors, observation endpoints, and sample sizes, etc. As a hematopoietic proteoglycan critically interacting with immune-stromal components, SRGN’s prognostic evaluation necessitates multivariate Cox regression models incorporating TME dynamics to resolve these platform- and molecular layer-dependent discrepancies.

Single-cell analysis revealed SRGN expression across 17 distinct cell subpopulations, with notable expression in dendritic cells, endothelial cells, macrophages, fibroblasts, mast cells, plasma cells, monocytes, T cells, and tumor-associated macrophages (TAMs). Immune infiltration analysis demonstrated a strong positive correlation between SRGN and most immune cell types, particularly macrophages (correlation coefficient of 0.7). SRGN expression was significantly elevated in macrophages within tumor samples compared to normal tissues, indicating its significant involvement in shaping the TME.

Gene Set Enrichment Analysis (GSEA) revealed SRGN associated with pathways like FCERI-mediated MAPK activation, cellular interactions at the vascular wall, JAK-STAT signaling, PI3K-AKT signaling, and PD-1 signaling, CTLA4 pathway. The FCERI-mediated MAPK activation pathway is crucial for immune cell activation and inflammatory responses (51–53), which are often hijacked by tumor cells to promote their growth and survival (10). The cellular interactions at the vascular wall pathway highlight SRGN’s potential role in angiogenesis and the formation of new blood vessels, which is essential for tumor expansion and metastasis (54). The JAK/STAT pathway is associated with inflammation, invasion, the formation of new blood vessels, metastasis, and the initiation and progression of cancer (55), while the PI3K-AKT signaling pathway is a key regulator of cell proliferation and survival (56). The PD-1 signaling and CTLA4 pathway are well-known immune checkpoint pathways that tumors often exploit to evade immune surveillance (57–60). Notably, macrophages can regulate their phagocytosis and antigen presentation function through PD-1/PD-L1 immune checkpoints, thereby promoting tumor cells to evade phagocytosis and clearance (58). Given that SRGN expression exhibits the highest positive correlation with macrophages among immune cells, this suggests that SRGN may enhance tumor immune evasion by modulating macrophage function via these checkpoints. Furthermore, immune infiltration analysis showed that SRGN expression is positively correlated with 40 immune checkpoint genes, further underscoring its role in immune modulation. The association of SRGN with these pathways suggests that it may contribute to tumor progression by modulating immune responses, promoting angiogenesis, and enhancing cell survival and proliferation. These findings provide insights into the potential mechanisms underlying SRGN’s pro-tumorigenic effects and its influence on the TME, particularly in relation to macrophages and VEGF-driven angiogenesis.

Our single-cell analyses revealed a marked upregulation of SRGN in macrophages, suggesting its role in modulating macrophage function within the tumor microenvironment. CellChat-based inference further indicated enhanced intercellular communication in tumors, particularly between macrophages and endothelial cells, with a pronounced enrichment in VEGF-related signaling. Since VEGF is a well-established driver of tumor angiogenesis and metastasis (61), and TAMs are recognized as key sources of pro-angiogenic factors including VEGF (62, 63), we hypothesized that SRGN may facilitate angiogenesis by augmenting VEGF signaling in the TME. To functionally validate this hypothesis, we performed co-culture experiments using SRGN-overexpressing hepatoma cells and macrophages. Significantly, both VEGFA mRNA and protein levels were substantially elevated in macrophages under SRGN-high conditions, confirming that SRGN potentiates VEGF-A production. These results provide direct experimental support for the VEGF network activation predicted by our CellChat analysis.

The present study showed that SRGN induced the formation of tubular structures in both HUVECs and HepG2 cells. The pro-angiogenic activity of SRGN was further confirmed in xenograft tumor tissues. High SRGN levels have been reported to promote the proliferation of HUVECs (64), and SRGN protein expression is positively correlated with vascular invasion in HCC patients (10, 65). However, vascular invasion had no effect on the prognostic potential of SRGN mRNA via the KM-plotter. Further studies are required to investigate this controversy.

By TIMER2.0 and GEPIA analysis, more crosstalk of SRGN and immune cells was indicated, especially monocyte/macrophage subsets. In addition to positive correlation between SRGN expression and so many immune checkpoint genes described above, SRGN levels were highest in M2 macrophages than other three monocyte/macrophage subsets in liver tissues, LIHC, and adjacent normal by GEPIA, reflecting the liver’s natural immune tolerance, immunosuppressive properties and pro-tumorigenic characteristics, respectively (66, 67). Even SRGN levels in M1 macrophages, which antagonize pro-tumorigenesis, were increased more in LIHC and adjacent normal than that in normal liver tissues.

Survival analysis revealed distinct immune contexts associated with SRGN expression. In the low-SRGN cohort, elevated macrophages (TIMER), M0 macrophages, and pro-tumorigenic M2 macrophages correlated with poor prognosis, suggesting a predominantly immunosuppressive microenvironment. Conversely, in the high-SRGN cohort, higher levels of monocytes (MCP-COUNTER) and nominally anti-tumorigenic M1 macrophages were unexpectedly associated with unfavorable outcomes. This suggests that high SRGN expression may drive a dysregulated hyperinflammatory state, potentially leading to immune exhaustion or functional impairment of M1 macrophages—shifting their role from antitumor to pro-tumor effect. It has reported that PD-L1 is induced in M1 macrophages through IL-1beta signaling (68). In the present study, SRGN showed positive correlation with both PD-L1 and IL-β (**Figure 3F**; **Figure 4C**; **Figure 9B**), implicating SRGN in facilitating immune escape and modulating response to immunotherapy. In summary, the prognostic significance of SRGN appears closely linked to the differentiation/polarization status and contextual behavior of monocyte-macrophage subsets within the TME.

*Using HepG2 cell line (which has low intrinsic tumorigenicity)*, *SRGN overexpression conferred proliferative advantage in vitro and tumorigenic capacity in vivo*, *directly establishing its pro-tumorigenic function in PLC.* SRGN expression can be induced during the differentiation of monocytes into macrophages or upon macrophage activation (8, 11). *The findings* were *confirmed* in the present *in vitro experiment*. SRGN was significantly upregulated by HepG2SG cells in supernatant of TAM2 which subsequently promoted invasion and migration of Huh7 and HepG2 cells. Subcutaneous xenografts derived from HepG2SG were heavier than the controls, with elevated CD206&^+^ staining in tumor tissues. Compared with TAM1, SRGN-overexpressing TAM2 showed higher CD206 expression, increased arginase1, and reduced NOS2 and IL-1β levels, while MMP9 showed no significant difference between TAM1 and TAM2. The protein levels of NF-κB, phospho-p65, and phospho-STAT3 were elevated while less than classical M2 macrophages. In glioblastoma, STAT3 phosphorylation was suppressed through SRGN knockdown (19); in contrast, STAT3 induced SRGN in nasopharyngeal carcinoma (69), suggesting a potential positive feedback loop in tumor cells that may also operate in TAMs, thereby sustaining a potent oncogenic signaling circuit. In addition, macrophages may be recruited through SRGN, as shown in myeloma (70) and disc degeneration (71). In conclusion, the results implied paracrine SRGN-driven M2-like polarization that may promote HCC progression.

Additionally, TAM populations exhibited a phenotypic heterogeneity. One of M1 markers CD80 was higher in TAM1 and TAM2 than classical M2 macrophages and no significant difference between the former two cell populations. But CD80 was reduced in HepG2SG mice, this contrasted with complexity of the in vivo TME.

Currently, no specific SRGN inhibitors are available, and related research has focused on combination strategies targeting its associated ligands and signaling molecules, e g., CD44 and YAP/TAZ (72). Although sorafenib with high binding affinity to SRGN (72), our experiments showed that sorafenib did not achieve significant inhibition in vitro or in subcutaneous tumor models, possibly due to variation among liver cancer cell types. Additionally, the glycosylation of SRGN and SRGN-related TME should be considered in targeted therapy development.

In the study, certain limitations need to be acknowledged. First, the bioinformatics analysis data mainly focus on hepatocellular carcinoma, yet the validation was conducted using the hepatoblastoma cell line HepG2, which has lower tumorigenicity in mice than Huh7, possibly affecting the accuracy and extrapolation of the results. Second, the study lacks some key macrophage markers like CD86 and CD163. CD86 is activated earlier than CD80, while CD80 and CD206 have similar expression timing, peaking between 48 and 72 h post-stimulation. The absence of these markers may lead to an incomplete understanding of macrophage polarization and function. Third, the mechanistic research is not fully comprehensive. There was no knockdown study on SRGN, and no antagonists and activators were used to validate the signal transduction pathways, making it difficult to confirm the specific pathways through which SRGN exerts its effects. Additionally, sequencing data and microarray analysis of tumor tissues may have systematic biases, and future research should incorporate more spatiotemporal single-cell RNA sequencing to verify the findings. In conclusion, SRGN is a limited prognostic factor in LIHC, this study comprehensively reveals the relationships between SRGN and immune cells, especially monocyte/macrophage subsets, which may contribute to the development of novel immunotherapy strategies.

## Data Availability

The datasets Analyzed for this study can be found in the GEO: Available at: GSE242889.

## References

[1] Ji F Zhang J Mao L Tan Y Ye M He X . Liver-specific gene PGRMC1 blocks c-Myc-induced hepatocarcinogenesis through ER stress-independent PERK activation. Nat Commun. (2025) 16:50. doi: 10.1038/s41467-024-55745-2, PMID: 39747098 PMC11696091

[2] Wang H Wu Z ui D Shi Y Zhai B . Radiofrequency ablation of hepatocellular carcinoma: Current status, challenges, and prospects. Liver research (Beijing, China). (2023) 7:108–115., PMID: 39958948 10.1016/j.livres.2023.05.002 PMC11791925

[3] Altekruse SF McGlynn KA Reichman ME . Hepatocellular carcinoma incidence, mortality, and survival trends in the United States from 1975 to 2005. J Clin Oncol. (2009) 27:1485–91. doi: 10.1200/JCO.2008.20.7753, PMID: 19224838 PMC2668555

[4] Fu MX Lambert G Cook A Ndow G Haddadin Y Shimakawa Y . Quality of life in patients with HBV infection: A systematic review and meta-analysis. JHEP Rep. (2025) 7:101312. doi: 10.1016/j.jhepr.2024.101312, PMID: 40115166 PMC11919624

[5] Chen F Li Q Xu X Wang F . Clinical characteristics and risk factors of hepatitis B virus-related cirrhosis/hepatocellular carcinoma: A single-center retrospective study. Liver research (Beijing, China). (2023) 7:237–243., PMID: 39958384 10.1016/j.livres.2023.07.004 PMC11791899

[6] Li B Yan C Zhu J Chen X Fu Q Zhang H . Anti-PD-1/PD-L1 blockade immunotherapy employed in treating hepatitis B virus infection-related advanced hepatocellular carcinoma: A literature review. Front Immunol. (2020) 11:1037. doi: 10.3389/fimmu.2020.01037, PMID: 32547550 PMC7270402

[7] Huang M Chen X Jiang Y Chan LWC . Kolmogorov-arnold network model integrated with hypoxia risk for predicting PD-L1 inhibitor responses in hepatocellular carcinoma. Bioengineering (Basel Switzerland). (2025) 12(3):322. doi: 10.3390/bioengineering12030322, PMID: 40150786 PMC11939538

[8] Stephenson EL Mishra MK Moussienko D Laflamme N Rivest S Ling CC . Chondroitin sulfate proteoglycans as novel drivers of leucocyte infiltration in multiple sclerosis. Brain. (2018) 141:1094–110. doi: 10.1093/brain/awy033, PMID: 29506186 PMC5888970

[9] Uhlin-Hansen L Eskeland T Kolset SO . Modulation of the expression of chondroitin sulfate proteoglycan in stimulated human monocytes. J Biol Chem. (1989) 264:14916–22. doi: 10.1016/S0021-9258(18)63789-5, PMID: 2768247

[10] He L Zhou X Qu C Tang Y Zhang Q Hong J . Serglycin (SRGN) overexpression predicts poor prognosis in hepatocellular carcinoma patients. Med Oncol (Northwood London England). (2013) 30:707. doi: 10.1007/s12032-013-0707-4, PMID: 23996242

[11] Kolset SO Pejler G . Serglycin: a structural and functional chameleon with wide impact on immune cells. J Immunol. (2011) 187:4927–33. doi: 10.4049/jimmunol.1100806, PMID: 22049227

[12] Zhang Y Li Z Chen C Wei W Li Z Zhou H . SRGN promotes macrophage recruitment through CCL3 in osteoarthritis. Connect Tissue Res. (2024) 65:330–42. doi: 10.1080/03008207.2024.2380313, PMID: 39067006

[13] Niemann CU Abrink M Pejler G Fischer RL Christensen EI Knight SD . Neutrophil elastase depends on serglycin proteoglycan for localization in granules. Blood. (2007) 109:4478–86. doi: 10.1182/blood-2006-02-001719, PMID: 17272511

[14] Grujic M Christensen JP Sørensen MR Abrink M Pejler G Thomsen AR . Delayed contraction of the CD8+ T cell response toward lymphocytic choriomeningitis virus infection in mice lacking serglycin. J Immunol. (2008) 181:1043–51. doi: 10.4049/jimmunol.181.2.1043, PMID: 18606656

[15] Chanzu H Lykins J Wigna-Kumar S Joshi S Pokrovskaya I Storrie B . Platelet α-granule cargo packaging and release are affected by the luminal proteoglycan, serglycin. J Thromb Haemost. (2021) 19:1082–95. doi: 10.1111/jth.15243, PMID: 33448622

[16] Korpetinou A Papachristou DJ Lampropoulou A Bouris P Labropoulou VT Noulas A . Increased expression of serglycin in specific carcinomas and aggressive cancer cell lines. BioMed Res Int. (2015) 2015:690721. doi: 10.1155/2015/690721, PMID: 26581653 PMC4637082

[17] He L Zhou X Qu C Tang Y Zhang Q Hong JJM . Serglycin (SRGN) overexpression predicts poor prognosis in hepatocellular carcinoma patients. (2013) 30:1–7., PMID: 23996242 10.1007/s12032-013-0707-4

[18] Korpetinou A Skandalis SS Labropoulou VT Smirlaki G Noulas A Karamanos NK . Serglycin: at the crossroad of inflammation and Malignancy. Front Oncol. (2014) 3:327. doi: 10.3389/fonc.2013.00327, PMID: 24455486 PMC3888995

[19] Manou D Bouris P Kletsas D Götte M Greve B Moustakas A . Serglycin activates pro-tumorigenic signaling and controls glioblastoma cell stemness, differentiation and invasive potential. Matrix Biol plus. (2020) 6-7:100033. doi: 10.1016/j.mbplus.2020.100033, PMID: 33543029 PMC7852318

[20] Tellez-Gabriel M Tekpli X Reine TM Hegge B Nielsen SR Chen M . Serglycin is involved in TGF-β Induced epithelial-mesenchymal transition and is highly expressed by immune cells in breast cancer tissue. Front Oncol. (2022) 12:868868. doi: 10.3389/fonc.2022.868868, PMID: 35494005 PMC9047906

[21] Tanaka I Dayde D Tai MC Mori H Solis LM Tripathi SC . SRGN-triggered aggressive and immunosuppressive phenotype in a subset of TTF-1-negative lung adenocarcinomas. J Natl Cancer Institute. (2022) 114:290–301. doi: 10.1093/jnci/djab183, PMID: 34524427 PMC8826620

[22] Zhu Y Lam AKY Shum DKY Cui D Zhang J Yan DD . Significance of serglycin and its binding partners in autocrine promotion of metastasis in esophageal cancer. Theranostics. (2021) 11:2722–41. doi: 10.7150/thno.49547, PMID: 33456569 PMC7806492

[23] Chu Q Huang H Huang T Cao L Peng L Shi S . Extracellular serglycin upregulates the CD44 receptor in an autocrine manner to maintain self-renewal in nasopharyngeal carcinoma cells by reciprocally activating the MAPK/β-catenin axis. Cell Death Dis. (2016) 7:e2456. doi: 10.1038/cddis.2016.287, PMID: 27809309 PMC5260886

[24] Yan D Cui D Zhu Y Chan CKW Choi CHJ Liu T . Serglycin-induced interleukin-1β from oesophageal cancer cells upregulate hepatocyte growth factor in fibroblasts to promote tumour angiogenesis and growth. Clin Trans Med. (2022) 12:e1031. doi: 10.1002/ctm2.1031, PMID: 35994394 PMC9394751

[25] Manou D Karamanos NK Theocharis AD . Tumorigenic functions of serglycin: Regulatory roles in epithelial to mesenchymal transition and oncogenic signaling. Semin Cancer Biol. (2020) 62:108–15. doi: 10.1016/j.semcancer.2019.07.004, PMID: 31279836

[26] Guo JY Chiu CH Wang MJ Li FA Chen JY . Proteoglycan serglycin promotes non-small cell lung cancer cell migration through the interaction of its glycosaminoglycans with CD44. J Biomed Sci. (2020) 27:2. doi: 10.1186/s12929-019-0600-3, PMID: 31898491 PMC6939340

[27] Li Y Zhu M Li G . Expression of serglycin in HepG2 cells with different HBV infection status. 111 RIVER ST, HOBOKEN 07030-5774, NJ USA: WILEY-BLACKWELL (2013) p. pp 914–914.

[28] Li Y Chen H Lu H Zou Z Li Y . Prognostic significance of hematopoietic-cell serglycin for the survival of hepatocellular carcinoma: A single-center retrospective study. Combinatorial Chem High throughput screening. (2021) 24:986–95. doi: 10.2174/1386207323666201020112459, PMID: 33081679

[29] Quan F Liang X Cheng M Yang H Liu K He S . Annotation of cell types (ACT): a convenient web server for cell type annotation. Genome Med. (2023) 15:91. doi: 10.1186/s13073-023-01249-5, PMID: 37924118 PMC10623726

[30] Li L Lu X Lian Q Wang X Jia C Xu C . Apoptotic vesicles of mesenchymal stem cells promote M2 polarization and alleviate early-onset preeclampsia via miR-191-5p. Stem Cell Res Ther. (2025) 16:414. doi: 10.1186/s13287-025-04546-5, PMID: 40739581 PMC12312347

[31] Huo X Jiang S Wu S Lian Q Chen H . Mechanosensitive ion channel-related genes in hepatocellular carcinoma: Unraveling prognostic genes and their roles in drug resistance and immune modulation. Liver Res (Beijing China). (2025) 9:36–48. doi: 10.1016/j.livres.2025.01.002, PMID: 40206431 PMC11977149

[32] Wang J Chen X Wu D Jia C Lian Q Pan Y . Single-cell and machine learning approaches uncover intrinsic immune-evasion genes in the prognosis of hepatocellular carcinoma. Liver Res (Beijing China). (2024) 8:282–94. doi: 10.1016/j.livres.2024.11.001, PMID: 39958919 PMC11771279

[33] Yang F Ni B Lian Q Qiu X He Y Zhang Q . Key genes associated with non-alcoholic fatty liver disease and hepatocellular carcinoma with metabolic risk factors. Front Genet. (2023) 14:1066410. doi: 10.3389/fgene.2023.1066410, PMID: 36950134 PMC10025510

[34] Hänzelmann S Castelo R Guinney J . GSVA: gene set variation analysis for microarray and RNA-seq data. BMC Bioinf. (2013) 14:7. doi: 10.1186/1471-2105-14-7, PMID: 23323831 PMC3618321

[35] Bindea G Mlecnik B Tosolini M Kirilovsky A Waldner M Obenauf AC . Spatiotemporal dynamics of intratumoral immune cells reveal the immune landscape in human cancer. Immunity. (2013) 39:782–95. doi: 10.1016/j.immuni.2013.10.003, PMID: 24138885

[36] Yang F Lian Q Ni B Qiu X He Y Zou X . MUTYH is a potential prognostic biomarker and correlates with immune infiltrates in hepatocellular carcinoma. Liver research (Beijing, China). (2022) 6:258–268., PMID: 39957908 10.1016/j.livres.2022.12.002 PMC11791856

[37] Tang Z Li C Kang B Gao G Li C Zhang Z . GEPIA: a web server for cancer and normal gene expression profiling and interactive analyses. Nucleic Acids Res. (2017) 45:W98–w102. doi: 10.1093/nar/gkx247, PMID: 28407145 PMC5570223

[38] Nagy Á. Munkácsy G Győrffy B . Pancancer survival analysis of cancer hallmark genes. Sci Rep. (2021) 11:6047. doi: 10.1038/s41598-021-84787-5, PMID: 33723286 PMC7961001

[39] Li T Fan J Wang B Traugh N Chen Q Liu JS . TIMER: A web server for comprehensive analysis of tumor-infiltrating immune cells. Cancer Res. (2017) 77:e108–10. doi: 10.1158/1538-7445.AM2017-108 PMC6042652 29092952

[40] Edwards NJ Oberti M Thangudu RR Cai S McGarvey PB Jacob S . The CPTAC data portal: A resource for cancer proteomics research. J Proteome Res. (2015) 14:2707–13. doi: 10.1021/pr501254j, PMID: 25873244

[41] Thul PJ Lindskog C . The human protein atlas: A spatial map of the human proteome. Protein science: Publ Protein Soc. (2018) 27:233–44. doi: 10.1002/pro.3307, PMID: 28940711 PMC5734309

[42] Cohen J . Statistical Power Analysis for the Behavioral Sciences (2nd ed.). Routledge: Taylor & Francis. (2013). Available online at: https://www.taylorfrancis.com/books/mono/10.4324/9780203771587/statistical-power-analysis-behavioral-sciences-jacob-cohen.

[43] Liu Q Lian Q Song Y Yang S Jia C Fang J . Identification of LSM family members as potential chemoresistance predictive and therapeutic biomarkers for gastric cancer. Front Oncol. (2023) 13:1119945. doi: 10.3389/fonc.2023.1119945, PMID: 37007092 PMC10064066

[44] Zhang Y Liang X Lian Q Liu L Zhang B Dong Z . Transcriptional analysis of the expression and prognostic value of lipid droplet-localized proteins in hepatocellular carcinoma. BMC Cancer. (2023) 23:677. doi: 10.1186/s12885-023-10987-z, PMID: 37464334 PMC10354995

[45] Li Y Li Y Zou Z Li Y Xie H Yang H . Yin Yang Gong Ji pill is an ancient formula with antitumor activity against hepatoma cells. J ethnopharmacology. (2020) 248:112267. doi: 10.1016/j.jep.2019.112267, PMID: 31586691

[46] Weidner N Semple JP Welch WR Folkman J . Tumor angiogenesis and metastasis–correlation in invasive breast carcinoma. New Engl J Med. (1991) 324:1–8. doi: 10.1056/NEJM199101033240101, PMID: 1701519

[47] Wang G Wu S Xiong Z Qu H Fang X Bao Y . CROST: a comprehensive repository of spatial transcriptomics. Nucleic Acids Res. (2024) 52:D882–d890. doi: 10.1093/nar/gkad782, PMID: 37791883 PMC10773281

[48] Wang X Xiong H Liang D Chen Z Li X Zhang K . The role of SRGN in the survival and immune infiltrates of skin cutaneous melanoma (SKCM) and SKCM-metastasis patients. BMC Cancer. (2020) 20:378. doi: 10.1186/s12885-020-06849-7, PMID: 32370744 PMC7201763

[49] Yang T Fu Z Zhang Y Wang M Mao C Ge W . Serum proteomics analysis of candidate predictive biomarker panel for the diagnosis of trastuzumab-based therapy resistant breast cancer. Biomedicine pharmacotherapy = Biomedecine pharmacotherapie. (2020) 129:110465. doi: 10.1016/j.biopha.2020.110465, PMID: 32887021

[50] Menyhárt O Nagy Á. Győrffy B . Determining consistent prognostic biomarkers of overall survival and vascular invasion in hepatocellular carcinoma. R Soc Open Sci. (2018) 5:181006. doi: 10.1098/rsos.181006, PMID: 30662724 PMC6304123

[51] Ayala TS Tessaro FHG Jannuzzi GP Bella LM Ferreira KS Martins JO . High glucose environments interfere with bone marrow-derived macrophage inflammatory mediator release, the TLR4 pathway and glucose metabolism. Sci Rep. (2019) 9:11447. doi: 10.1038/s41598-019-47836-8, PMID: 31391499 PMC6686006

[52] Lee JY Kang SR Yune TY . Fluoxetine prevents oligodendrocyte cell death by inhibiting microglia activation after spinal cord injury. J neurotrauma. (2015) 32:633–44. doi: 10.1089/neu.2014.3527, PMID: 25366938 PMC4410451

[53] Bo N Yilin H Chaoyue Y Lu L Yuan Y . Acrylamide induces NLRP3 inflammasome activation via oxidative stress- and endoplasmic reticulum stress-mediated MAPK pathway in HepG2 cells. Food Chem Toxicol. (2020) 145:111679. doi: 10.1016/j.fct.2020.111679, PMID: 32805340

[54] Khan K Long B Deshpande GM Fox PL . Bidirectional tumor-promoting activities of macrophage ezrin. Int J Mol Sci. (2020) 21(20):7716. doi: 10.3390/ijms21207716, PMID: 33086476 PMC7589996

[55] Johnson RP Ratnacaram CK Kumar L Jose J . Combinatorial approaches of nanotherapeutics for inflammatory pathway targeted therapy of prostate cancer. Drug resistance updates. (2022) 64:100865. doi: 10.1016/j.drup.2022.100865, PMID: 36099796

[56] Mause SF Ritzel E Liehn EA Hristov M Bidzhekov K Müller-Newen G . Platelet microparticles enhance the vasoregenerative potential of angiogenic early outgrowth cells after vascular injury. Circulation. (2010) 122:495–506. doi: 10.1161/CIRCULATIONAHA.109.909473, PMID: 20644015

[57] Ali MHM Toor SM Rakib F Mall R Ullah E Mroue K . Investigation of the effect of PD-L1 blockade on triple negative breast cancer cells using fourier transform infrared spectroscopy. Vaccines. (2019) 7(3):109. doi: 10.3390/vaccines7030109, PMID: 31505846 PMC6789440

[58] Fang W Zhou T Shi H Yao M Zhang D Qian H . Progranulin induces immune escape in breast cancer via up-regulating PD-L1 expression on tumor-associated macrophages (TAMs) and promoting CD8(+) T cell exclusion. J Exp Clin Cancer research: CR. (2021) 40:4. doi: 10.1186/s13046-020-01786-6, PMID: 33390170 PMC7780622

[59] Yu Y Tang H Franceschi D Mujagond P Acharya A Deng Y . Immune checkpoint gene expression profiling identifies programmed cell death ligand-1 centered immunologic subtypes of oral and squamous cell carcinoma with favorable survival. Front Med. (2021) 8:759605. doi: 10.3389/fmed.2021.759605, PMID: 35127742 PMC8810827

[60] van den Boorn JG Hartmann G . Turning tumors into vaccines: co-opting the innate immune system. Immunity. (2013) 39:27–37. doi: 10.1016/j.immuni.2013.07.011, PMID: 23890061

[61] Kuol N Stojanovska L Apostolopoulos V Nurgali K . Role of the nervous system in cancer metastasis. J Exp Clin Cancer research: CR. (2018) 37:5. doi: 10.1186/s13046-018-0674-x, PMID: 29334991 PMC5769535

[62] Navakanitworakul R Hung WT Gunewardena S Davis JS Chotigeat W Christenson LK . Characterization and small RNA content of extracellular vesicles in follicular fluid of developing bovine antral follicles. Sci Rep. (2016) 6:25486. doi: 10.1038/srep25486, PMID: 27158133 PMC4860563

[63] Giavazzi R Sennino B Coltrini D Garofalo A Dossi R Ronca R . Distinct role of fibroblast growth factor-2 and vascular endothelial growth factor on tumor growth and angiogenesis. Am J Pathol. (2003) 162:1913–26. doi: 10.1016/S0002-9440(10)64325-8, PMID: 12759248 PMC1868139

[64] Ma Q Gu W Li T Zhang K Cui Y Qu K . SRGN, a new identified shear-stress-responsive gene in endothelial cells. Mol Cell Biochem. (2020) 474:15–26. doi: 10.1007/s11010-020-03830-7, PMID: 32712749

[65] Zahran AM Abdel-Rahim MH Refaat A Sayed M Othman MM Khalak LMR . Circulating hematopoietic stem cells, endothelial progenitor cells and cancer stem cells in hepatocellular carcinoma patients: contribution to diagnosis and prognosis. Acta Oncol (Stockholm Sweden). (2020) 59:33–9. doi: 10.1080/0284186X.2019.1657940, PMID: 31478425

[66] Sutton VR Brennan AJ Ellis S Danne J Thia K Jenkins MR . Serglycin determines secretory granule repertoire and regulates natural killer cell and cytotoxic T lymphocyte cytotoxicity. FEBS J. (2016) 283:947–61. doi: 10.1111/febs.13649, PMID: 26756195

[67] Guidotti LG Inverso D Sironi L Di Lucia P Fioravanti J Ganzer L . Immunosurveillance of the liver by intravascular effector CD8(+) T cells. Cell. (2015) 161:486–500. doi: 10.1016/j.cell.2015.03.005, PMID: 25892224 PMC11630812

[68] Zong Z Zou J Mao R Ma C Li N Wang J . M1 macrophages induce PD-L1 expression in hepatocellular carcinoma cells through IL-1β Signaling. Front Immunol. (2019) 10:1643. doi: 10.3389/fimmu.2019.01643, PMID: 31379842 PMC6648893

[69] Wang YL Ren D Lu JL Jiang H Wei JZ Lan J . STAT3 regulates SRGN and promotes metastasis of nasopharyngeal carcinoma through the FoxO1-miR-148a-5p-CREB1 axis. Lab investigation; J Tech Methods Pathol. (2022) 102:919–34. doi: 10.1038/s41374-022-00733-7, PMID: 35562411

[70] Cheng K Cai N Zhu J Yang X Liang H Zhang W . Tumor-associated macrophages in liver cancer: From mechanisms to therapy. Cancer Commun (London England). (2022) 42:1112–40. doi: 10.1002/cac2.12345, PMID: 36069342 PMC9648394

[71] Chen F Lei L Chen S Zhao Z Huang Y Jiang G . Serglycin secreted by late-stage nucleus pulposus cells is a biomarker of intervertebral disc degeneration. Nat Commun. (2024) 15:47. doi: 10.1038/s41467-023-44313-9, PMID: 38167807 PMC10761730

[72] Zhang S Hu H Li X Chen Q Zheng Y Peng H . SRGN-mediated reactivation of the YAP/CRISPLD2 axis promotes aggressiveness of hepatocellular carcinoma. Int J Biol Sci. (2025) 21:3262–85. doi: 10.7150/ijbs.108151, PMID: 40384866 PMC12080381

