# Supplementary material for: Serglycin’s role in primary liver cancer: insights into tumor microenvironment and macrophage interaction
Source: Front Immunol. 2025 Dec 16;16:1668627. doi: 10.3389/fimmu.2025.1668627 (PMC12748857; doi:10.3389/fimmu.2025.1668627)
Supplement: Supplementary file 1 [file DataSheet1.docx]

**Supplementary materials**

**Serglycin's Role in Primary Liver Cancer: Insights into Tumor Microenvironment and Macrophage Interaction**

Qinghai Lian^1,2,3,+^, Chonghan Ma^1,2,3,+^, Xiaoxiao Wang^6^*^+^*, Jiani Wang^4^, Jindi Zeng^5*^, Jiongshan Zhang^6*^, Yongwei Li^6*^

1Vaccine Research Institute, Sun Yat-Sen University, Guangzhou, Guangdong, China,

2Cell-Gene Therapy Translational Medicine Research Centre, The Third Affiliated Hospital, Sun Yat-Sen University, Guangzhou, Guangdong, China,

3Biotherapy Center, The Third Affiliated Hospital, Sun Yat-Sen University, Guangzhou, Guangdong, China, 4Department of Thyroid and Breast Surgery, The Third Affiliated Hospital of Sun Yat-sen University, Guangzhou, Guangdong, China,

5Department of Stomatology, First Affiliated Hospital of Guangzhou Medical University, Guangzhou, Guangdong, China,

6Department of Traditional Chinese Medicine, The Third Affiliated Hospital of Sun Yat-sen University,

Guangzhou, Guangdong, China

**+ Qinghai Lian, Chonghan Ma, and Xiaoxiao Wang are co-first authors.**

**Corresponding authors**

*Correspondence to Yongwei Li (liyongw@mail.sysu.edu.cn), Jiongshan Zhang (zhjshan2@ mail.sysu.edu.cn), or Jindi Zeng ([zenjindi@126.com](mailto:zenjindi@126.com)).


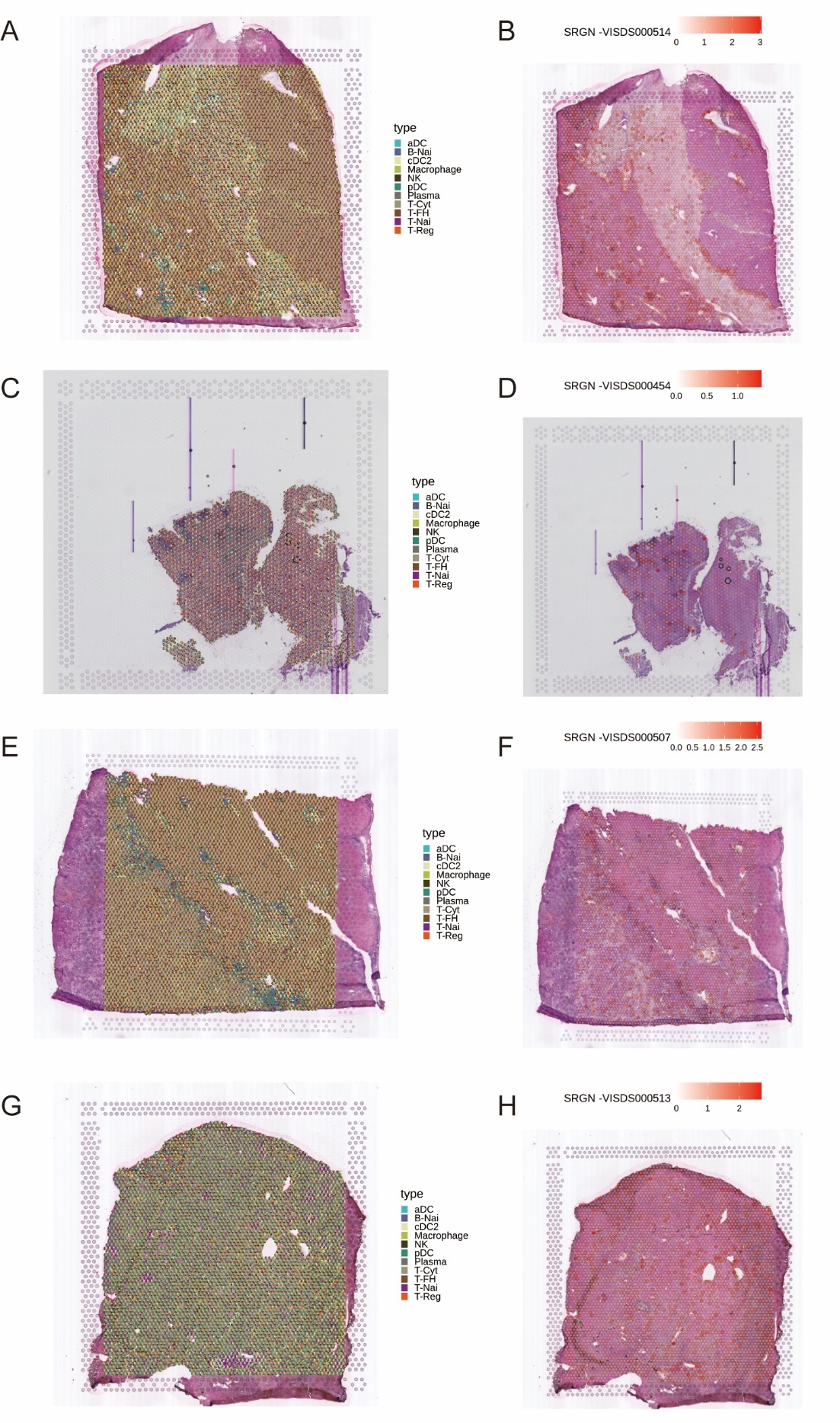


Figure S1 Spatial distribution of cell types in HCC tissues across multiple samples: VISDS000514 (A), VISDS000454 (C), VISDS000507 (E), and VISDS000513 (G). Spatial localization and gene expression dynamics of SRGN in VISDS000514 (B), VISDS000454 (D), VISDS000507 (F) and VISDS000513 (H).


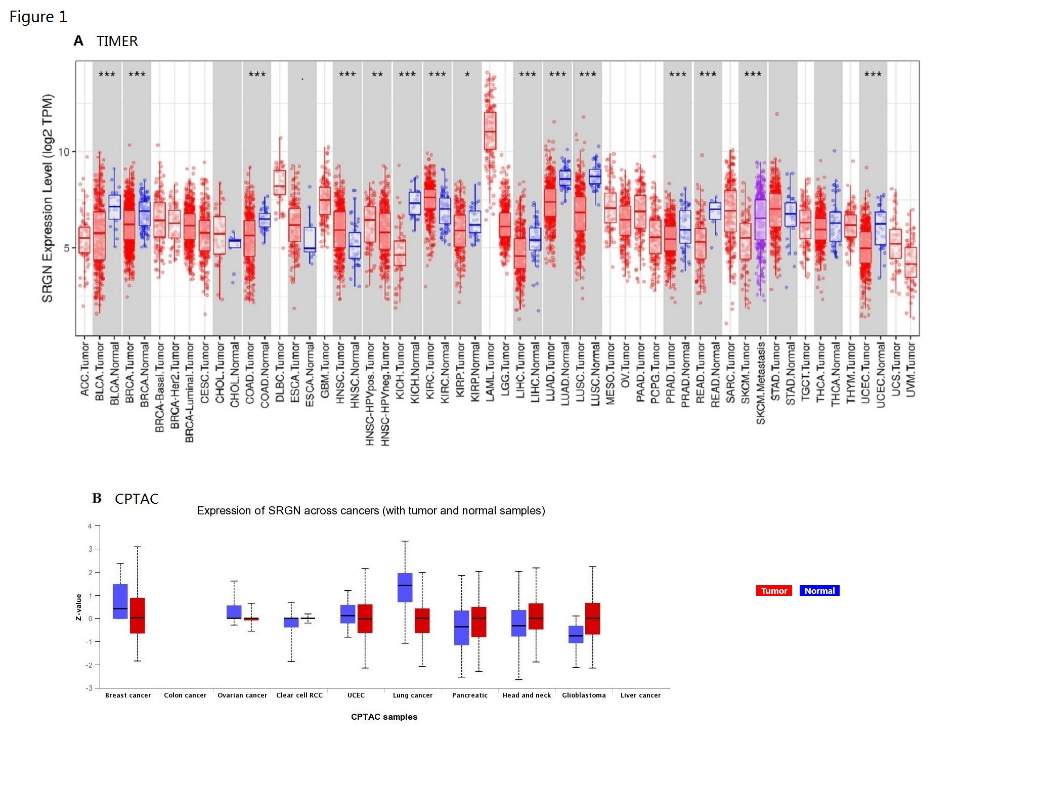


**Figure S2** A) Differential expression of SRGN in tumor (red column) compared with adjacent normal tissues (blue column) across all TCGA tumors by Wilcoxon’s test using TIMER (Diff Exp module) (^*^*P* < 0.05, ^**^*P* < 0.01, ^***^*P* < 0.001). (B) The University of ALabama at Birmingham CANcer data analysis (UALCAN) portal provides protein expression analysis using data from the Clinical Proteomic Tumor Analysis Consortium (CPTAC). Protein expression data for liver cancer are not available.


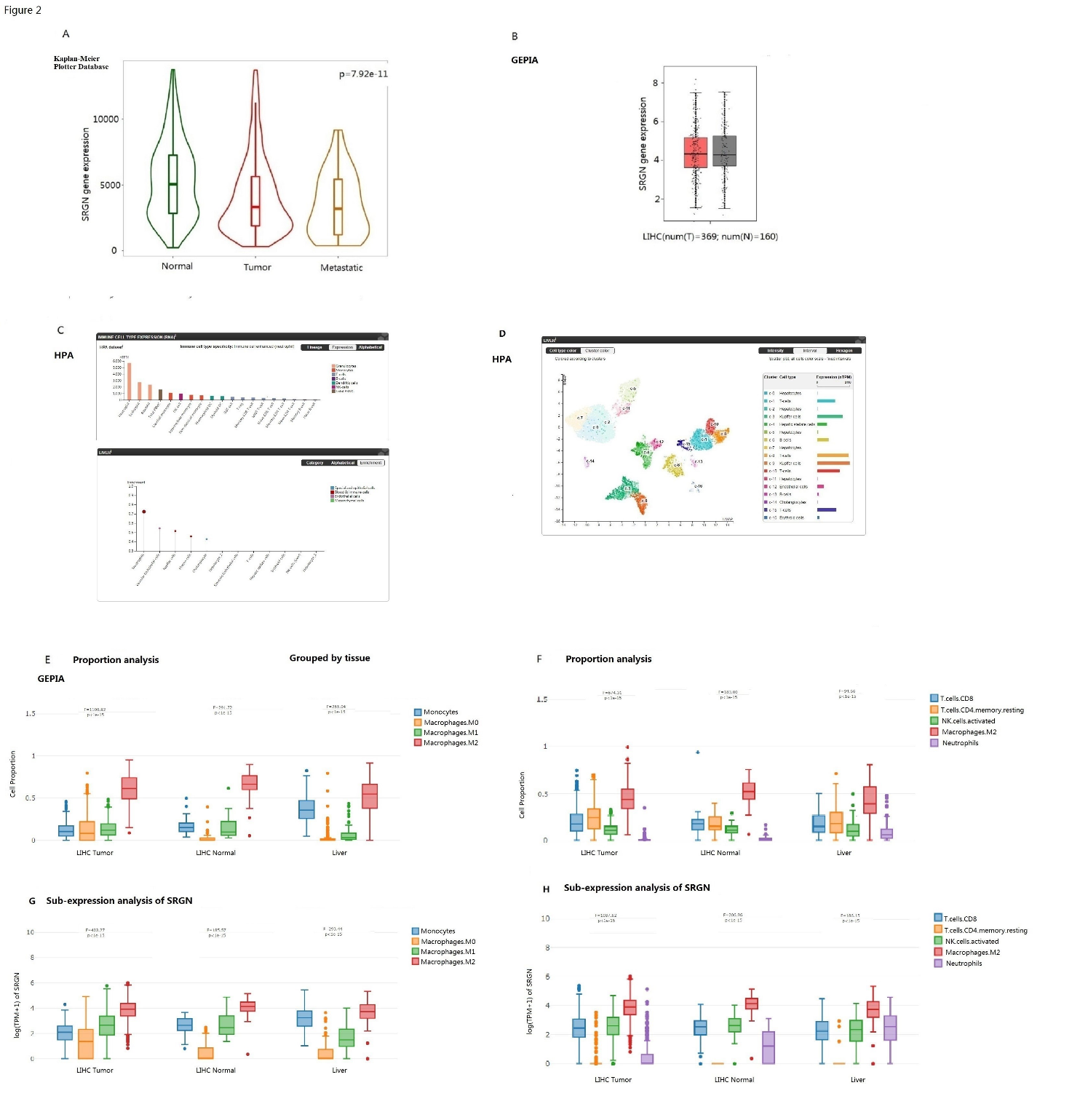


**Figure S3** (A) SRGN gene expression in tumor, adjacent normal, and metastatic liver cancer tissues via the Kaplan–Meier plotter database. (B) Expression of SRGN RNA in LIHC and adjacent normal tissues via GEPIA. (C) SRGN RNA was expressed in immune cell types in the order neutrophils > eosinophils >basophils > total peripheral blood mononuclear cells > classical monocytes, and in liver cell types in the order neutrophils >endothelial cells > Kupffer cells > plasma cells > cholangiocytes via The Human Protein Atlas (HPA). (D) SRGN RNA in liver cells via HPA. SRGN was prominently expressed in T cells, Kupffer cells, B cells, hepatic stellate cells, and endothelial cells, and barely expressed in hepatocytes. (E, F) GEPIA proportion analysis. The proportion of each cell type shown with interactive boxplots by ANOVA test among cell types or TCGA/GTEx sub-datasets. (G, H) GEPIA sub-expression analysis. SRGN expression in each cell type shown with interactive boxplot by ANOVA test. All cell types were analyzed, but only cells with strong correlations with SRGN or prognostic potential are shown.


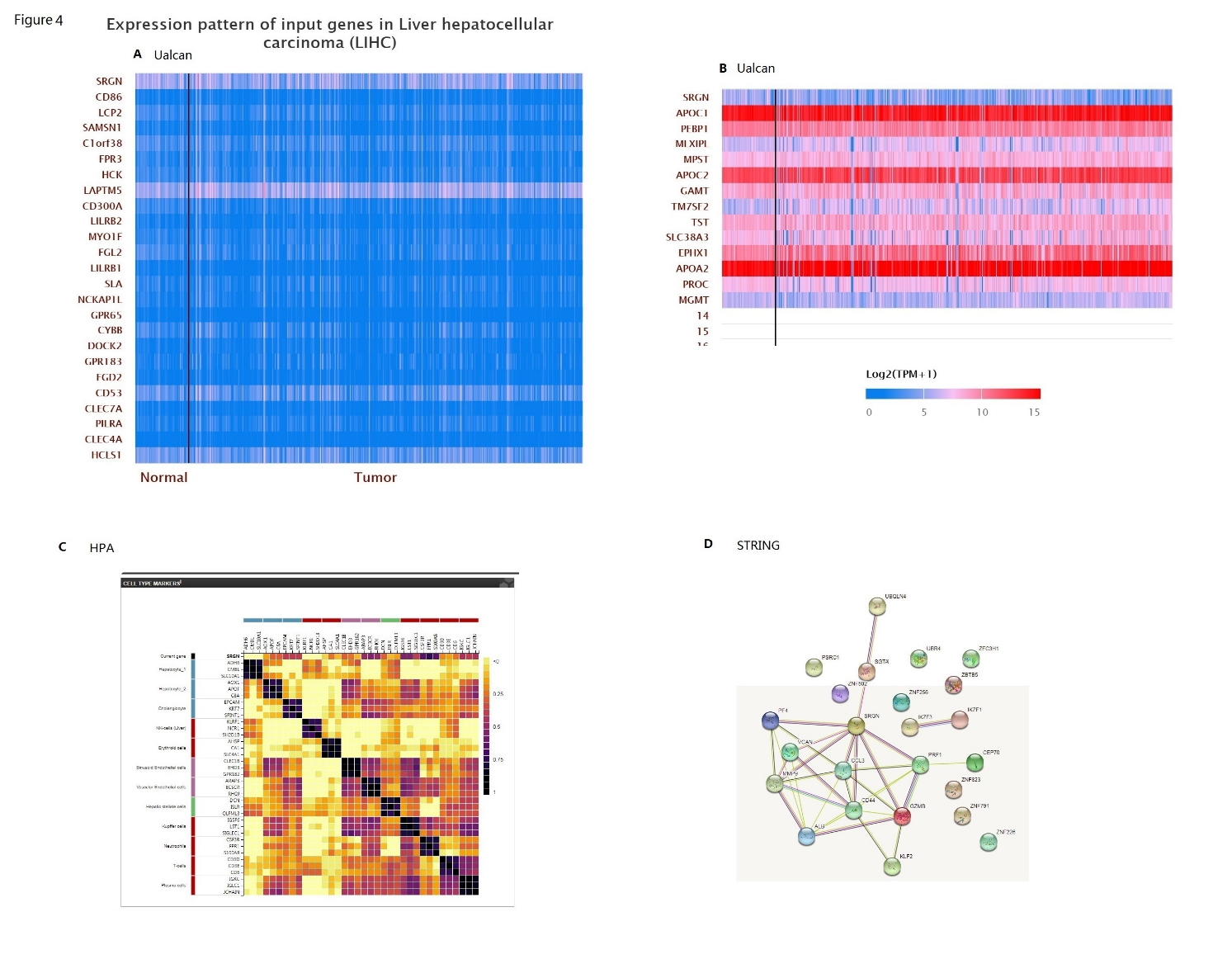


**Figure S4** Genes positively (A) and negatively (B) correlated with SRGN in LIHC by UALCAN analysis (data from TCGA). (C) Correlations between SRGN and liver cell biomarkers via HPA. (D) Predicted functional proteins with SRGN (Homo sapiens) by STRING analysis.


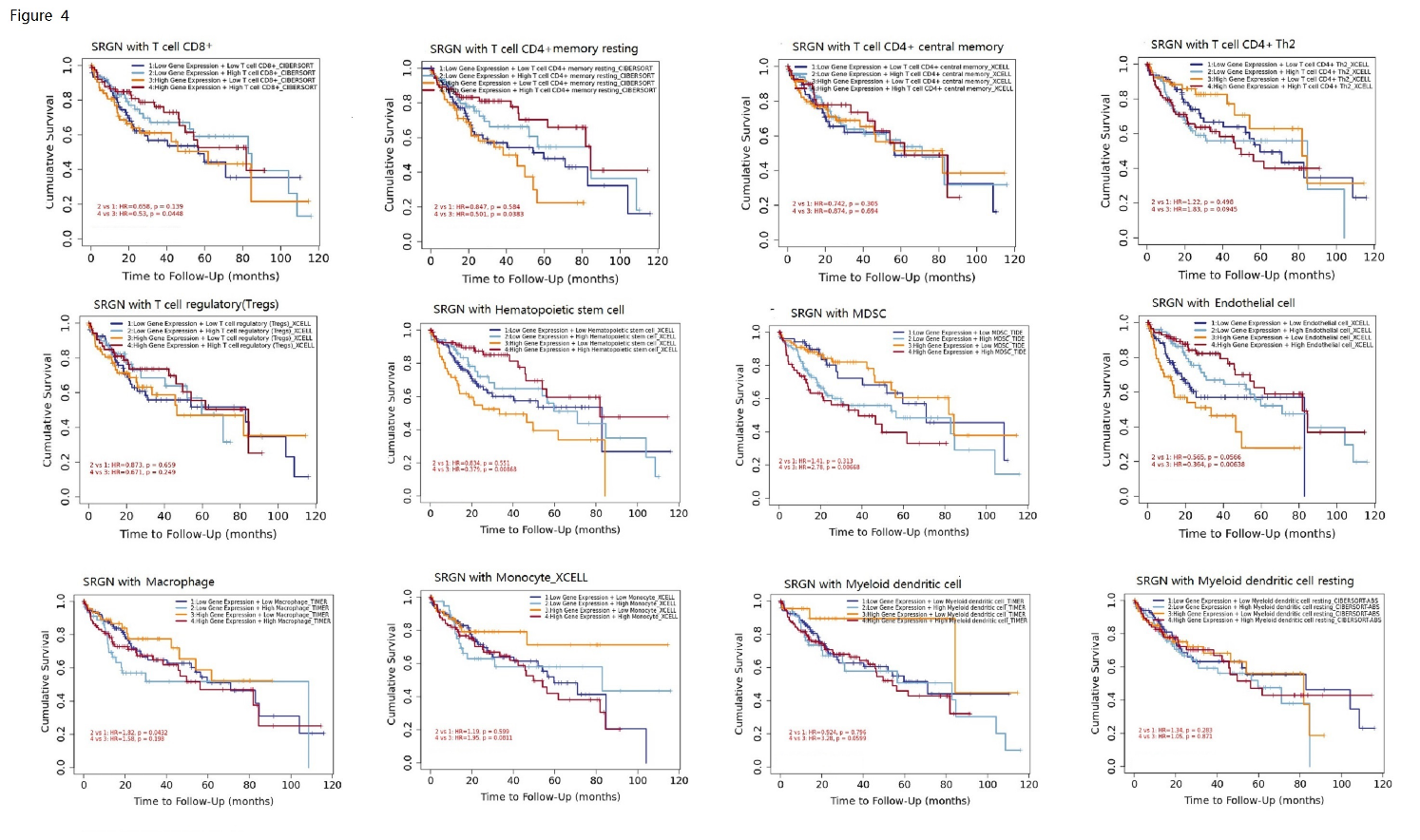


**Figure S5** Comparisons of Kaplan–Meier survival curves between cohorts with high and low levels of SRGN expression associated with high and low levels of infiltrating cells in LIHC via TIMER2.0.


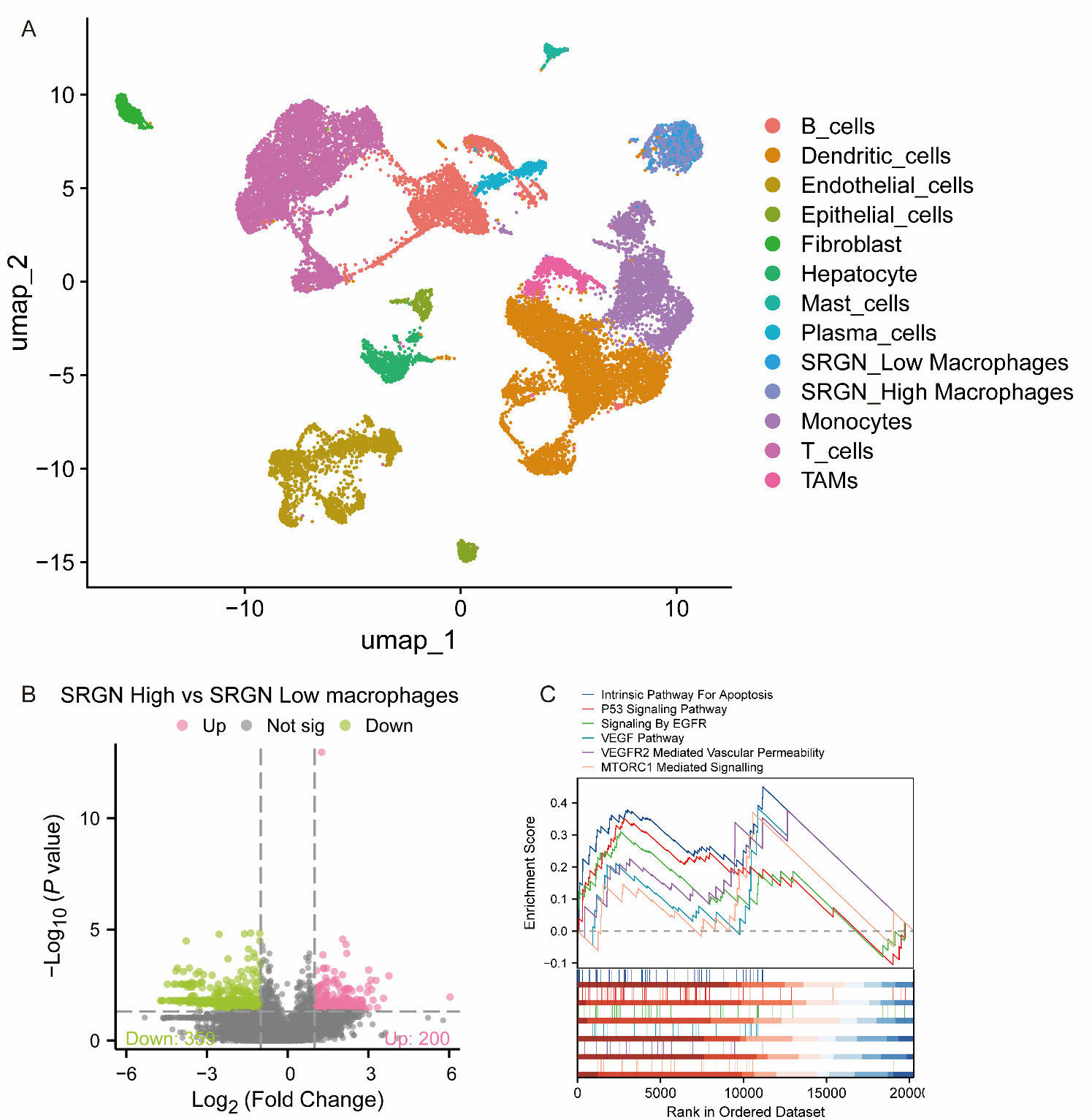


Figure S6 Functional implications of SRGN expression in macrophages. (A) UMAP visualization of macrophages, visually distinguished following reclassification into SRGN -High and SRGN -Low subgroups. (B) Volcano plot showing differential gene expression between SRGN_High and SRGN_Low macrophages (split by median SRGN expression). (C) Gene set enrichment analysis (GSEA) of transcriptional differences between SRGN -High and SRGN -Low macrophages.


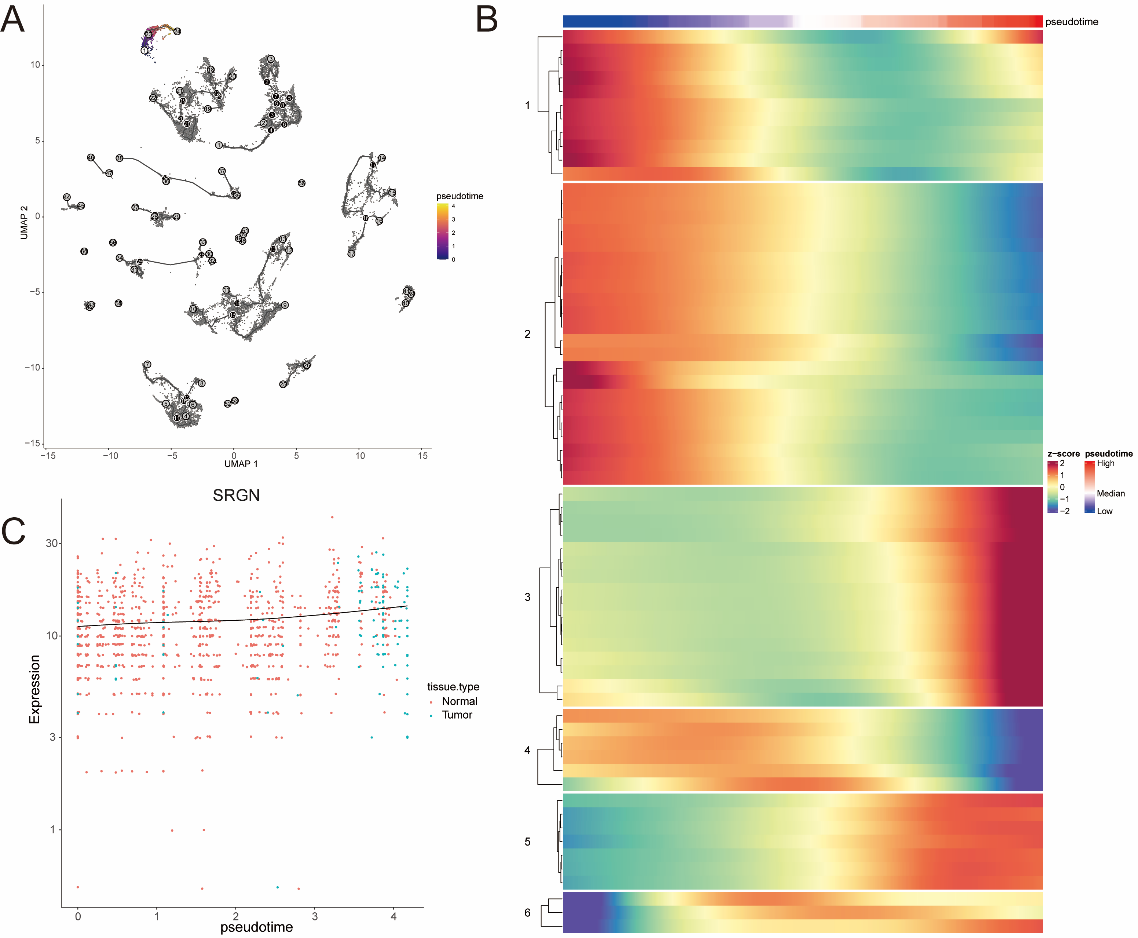


Figure S7 Pseudotime analysis. (A) Trajectory analysis by Monocle3. (B) Pseudotime heatmap for macrophage pseudotime trajectory analysis showing 6 differentially expressed gene clusters. (C) Expression dynamics of SRGN along the macrophage pseudotime trajectory.

Table1 The correlation of serglycin expression and prognosis in cancers via four bioinformatics databases

| Database and cancer | ID | Patients | Survival | Hazard ratio(HR) | *p*-value |
| --- | --- | --- | --- | --- | --- |
| GEPIA^#^ | ID | Sample size | Survival | HR(high) | p-value |
| TGCT  GBM  SKCM  SKCM | NA  NA  NA  NA | 68/68  80/81  228/229  228/229 | OS  RFS  OS  RFS | 9.1e8  1.7  0.51  0.74 | 0.022  0.013  5.2e-07  0.014 |
| TIMER2.0 | ID | Sample size | Survival | HR | p-value |
| SARC  SKCM  SKCM metastasis  ACC  LGG | NA  NA  NA  NA  NA | 260  471  368  79  516 | OS  OS  OS  OS  OS | 0.762  0.746  0.734  1.49  1.19 | 0.0213  0.000873  0.00184  0.0659  0.0691 |
| Kaplan-Meier plotter^*^ | ID | Sample size | Survival | HR(95%CI) | p-value |
| Breast cancer | 201859 _at  201859_at  201858_s_at  201858_s_at | 458  4929  1879  4929 | PPS  RFS  OS  RFS | 1.39( 1.09-1.76)  1.15(1.03-1.27)  0.74(0.61-0.91)  1.13(1.01-1.27) | 0.0066  0.012  0.0038  0.034 |
| Ovarian cancer | 201858_s_at  201859_at  201859_at  201859_at | 1435  1435  1656  782 | PFS  PFS  OS  PPS | 1.26,(1.11-1.45)  1.3(1.12-1.5)  1.15(1-1.33)  1.21(1-1.46) | 0.00054  0.00038  0.051  0.05 |
| Lung cancer | 201859_at  201859_at  201858_s_at | 982  1925  1925 | FP  OS  OS | 0.58(0.48- 0.71)  0.78 (0.69 -0.89)  1.25(1.1-1.42) | 9.9e-08  0.00022  0.00081 |
| Gastric cancer | 201858_s_at  201858_s_at  201858_s_at  201859_at  201859_at  201859_at | 875  498  640  640  875  498 | OS  PPS  FP  FP  OS  PPS | 0.55(0.46-0.66)  0.49(0.38-0.62)  0.57(0.47-0.7)  0.57(0.46-0.7）  0.58(0.49-0.69)  0.56(0.44-0.7) | 6.3e-12  9.7e-10  4.8e-08  1e-07  2.1e-10  3.8e-07 |
| Liver cancer | 5552  5552  5552  5552 | 366  364  313  357 | PFS  OS  RFS  DSS | 0.67( 0.5-0.91)  0.68(0.47-0.97)  0.65((0.46-0.92)  0.58(0.35-0.97) | 0.0095  0.034  0.015  0.034 |

The survival analysis of *SRGN* expression in pan-cancer. NA: the data was not shown; *Split patients by auto select best cutoff, other factors such as pathology, risk factors, patient, and follow up threshold, etc., were chosen all; ^#^group cutoff: median, cutoff high/low: 50%. LGG, brain lower grade glioma; ACC, adrenocortical carcinoma; SKCM, skin cutaneous melanoma; TGCT, testicular germ cell tumor; SARC, sarcoma; GBM, glioblastoma multiforme; OS, overall survival; PFS, progression free survival; RFS, relapse-free survival; PPS, post progression survival; FP, first progression; DSS, disease-specific survival; DMFS, distant metastasis-free survival; EFS, event free survival.

**Table2. The correlation between SRGN and infiltrating cells in LIHC by TIMER2.0**

| TME Cells | Rho | *p*-value |
| --- | --- | --- |
| T cell CD4+  T cell CD4+ naïve_CIBERSORT)  T cell CD4+ naïve_XCELL)  T cell CD4+ central memory  T cell CD4+ effector memory  T cell CD4+ memory activated  T cell CD4+ memory resting  T cell CD4+ memory  T cell CD4+(non-regulatory)  T cell CD4+ Th1  T cell CD4+ Th2 | 0.368  -0.152  0.171  -0.262  0.107  0.12  0.412  0.305  0.175  -0.234  0.206 | 1.76e-12  4.69e-03  1.44e-03  7.79e-07  4.8e-02  2.64e-02  1.55e-15  7.2e-09  1.1e-03  1.09e05  1.19e-04 |
| T cell CD8+  T cell CD8+ central memory  T cell CD8+ effector memory | **0.552**  0.336  0.119 | **5.79e-29**  1.49e-10  2.65e-02 |
| B cell  B cell naïve  B cell plasma_CIBERSORT-ABS  B cell plasma_XCELL  B cell memory | 0.308  0.145  0.121  -0.11  0.108 | 5.19e-09  7.06e-03  2.4e-02  4.12e-02  4.49e-02 |
| T cell regulatory | 0.399 | 1.34e**-**14 |
| T cell follicular helper | 0.252 | 2.22e-06 |
| Monocyte  Macrophage/monocyte  Macrophage_EPIC  Macrophage_TIMER  Macrophage M0  Macrophage M1  Macrophage M2_CIBERSORT-ABS  Macrophage M2_TIDE | 0.481  0.481  -0.382  0.481  -0.123  **0.517**  **0.696**  **-0.516** | 2.21e-21  2.21e-21  1.9e-13  2.31e-21  2.23e-02  **5.84e-25**  **3.26e-51**  **6.66e-25** |
| Myloid dendritic cell  Myloid dendritic cell activated  Myloid dendritic cell resting | **0.627**  0.475  0.142 | **4.97e-39**  7.62e-21  8.13e-03 |
| Common lymphoid progenitor | 0.211 | 8.13e-05 |
| Neutrophil | 0.426 | 1.11e-16 |
| NK cell  NK cell resting  NK cell activated | -0.115  -0.164  **0.524** | 3.26e-02  2.18e-03  **1.11e-25** |
| T cell NK | -0.147 | 6.11e-03 |
| Mast cell  Mast cell resting  Mast cell activated | 0.109  0.192  -0.166 | 4.27e-02  3.23e-04  2.03e03 |
| Endothelial cell | **0.522** | **1.67e-25** |
| Cancer associated fibroblast | 0.456 | 4.09e-19 |

**Table 3. The correlation between SRGN and gene markers of infiltrating cells in LIHC by TIMER2.0**

| Gene markers | Rho | p-value |
| --- | --- | --- |
| CCL2  CD68  CD163  CD206  CSF1R  MS4A4A  PTGS2  VSIG4  IL-10  CD274  CTLA4  PDCD1  CD44  VEGFC  TGFβ2 | 0.482  0.552  0.757  0.567  0.724  0.757  0.574  0.649  0.597  0.65  0.45  0.43  0.527  0.485  0.437 | 1.77e-21  6.31e-29  2.29e-65  6.32e-33  3e-57  2.53e-65  1.17e-31  1.06e-42  9.32e-35  9.99e-43  1.3e-18  5.45e-17  4.33e-26  9.71e-22  1.55e-17 |
| CD8A  CD8B  CD86  NOS2  CD80  IRF5 | **0.566**  0.46  0.782  0.17  0.697  0.247 | **1.42e-30**  1.67e-19  1.64e-72  1.54e-04  1.47e-51  3.36e-06 |

**Table 4. Correlation of *SRGN* mRNA expression and prognosis in liver cancer with different clinical factors by**

**Kaplan-Meier plotter.**

| Clinical characteristics^*^ | N | OS (n= 364)  Hazard ratio | *p*-value | N | RFS(n=313)  Hazard ratio | *p*-value |
| --- | --- | --- | --- | --- | --- | --- |
| Male  Female | 246  118 | 0.56(0.36-0.88)  0.49(0.24-0.99) | **0.01**  **0.044** | 208  105 | 0.56(0.36-0.88)  0.56(0.28-1.1) | **0.0051**  0.085 |
| White  Asian | 181  155 | 0.58(0.36-0.93)  0.51(0.28-0.94) | **0.022**  **0.028** | 147  143 | 0.75(0.48-1.18)  0.55(0.33-0.92) | 0.21  **0.022** |
| Hepatitis virus(yes)  (no) | 150  167 | 0.72(0.38-1.37)  0.39(0.23-0.64) | 0.31  **0.00014** | 138  142 | 0.53(0.31-0.88)  0.65(0.38-1.12) | **0.014**  0.12 |
| Alcohol consumption(yes)  (no) | 115  202 | 0.35(0.18-0.7)  0.65(0.38-1.1) | **0.0017**  0.1 | 98  182 | 0.52(0.28-0.97)  0.63(0.38-1.02) | **0.037**  0.06 |
| Sorafenib treatment | 29 | 0.51(0.17-1.54) | 0.23 | 22 | 0.41(0.16-1.07) | 0.06 |
| Vascular invasion(none)  (micro) | 203  90 | 0.65(0.38-1.12)  2.14(0.74-6.22) | 0.12  0.15 | 175  81 | 0.74(0.45-1.21)  0.59(0.27-1.3) | 0.23  0.18 |
| AJCC_T(1)  (2)  (3) | 180  90  78 | 0.76(0.42-1.39)  0.44(0.21-0.95)  0.46(0.23-0.9) | 0.37  **0.031**  **0.021** | 160  79  65 | 1.27(0.72-2.24)  0.46(0.21-1.01)  0.56(0.27-1.15) | 0.4  **0.046**  0.11 |
| Grade (1)  2  3 | 55  174  118 | 0.44(0.17-1.14)  0.55(0.33-0.94)  1.3(0.69-2.46) | 0.084  **0.026**  0.42 | 45  147  106 | 1.74(0.64-4.71)  0.53(0.31-0.93)  0.54(0.31-0.93) | 0.27  **0.024**  **0.025** |
| Stage 1  1+2  2  2+3  3  3+4 | 170  253  83  166  83  87 | 0.73(0.39-1.38)  0.67(0.42-1.09)  0.45(0.2-1.01)  0.49(0.3-0.79)  0.41(0.21-0.8)  0.45(0.24-0.85) | 0.34  0.11  **0.046**  **0.0028**  **0.0071**  **0.011** | 153  227  74  142  68  68 | 1.55(0.87-2.76)  0.74(0.48-1.13)  0.51(0.25-1.05)  0.52(0.33-0.81)  0.54(0.29-1.01)  0.54(0.29-1.01) | 0.14  0.16  0.063  **0.0033**  0.051  0.051 |
| Clinical characteristics | N | PFS (n=366)  Hazard ratio | p-value | N | DSS(n=357)  Hazard ratio | p-value |
| Male  Female | 246  120 | 0.54（0.37-0.77）  0.56（0.31-1.01） | **0.00073**  0.051 | 241  116 | 0.53（0.29-0.97）  0.41（0.16-1.08） | **0.037**  0.062 |
| White  Asian | 183  155 | 0.73（0.49-1.07）  0.58（0.36-0.94） | 0.11  **0.025** | 177  152 | 0.62（0.35-1.11）  0.31（0.14-0.68） | 0.1  **0.002** |
| Hepatitis virus(yes)  (no) | 152  167 | 0.55（0.34-0.88）  0.64（0.39-1.03） | **0.011**  0.062 | 149  163 | 1.54（0.67-3.55）  0.37（0.2-0.69） | 0.31  **0.0013** |
| Alcohol consumption(yes)  (no) | 115  204 | 0.43（0.25-0.74）  0.68（0.43-1.08） | **0.002**  0.098 | 115  197 | 0.29（0.13-0.66）  0.63（0.31-1.29） | **0.0017**  0.2 |
| Sorafenib treatment | 30 | 0.37（0.15-0.9） | **0.023** | 29 | 0.51（0.17-1.54） | 0.23 |
| Vascular invasion(none)  (micro) | 204  91 | 0.84（0.54-1.3）  0.61（0.34-1.08） | 0.43  0.084 | 200  88 | 1.46（0.7-3.04）  4.62(0.6-35.68) | 0.31  0.11 |
| AJCC_T(1)  (2)  (3) | 180  92  78 | 1.58（0.95-2.63）  0.45（0.25-0.8）  0.46（0.24-0.88） | 0.073  **0.0049**  **0.016** | 177  89  75 | 3.82(0.9-16.26)  0.36(0.13-1.04)  0.4(0.18-0.89) | 0.051  **0.049**  **0.021** |
| Grade (1)  2  3 | 55  175  119 | 0.4（0.18-0.9）  0.6（0.37-0.96）  0.54（0.32-0.93） | **0.023**  **0.031**  **0.024** | 55  169  116 | 0.32(0.09-1.17)  0.48(0.24-0.95)  1.31(0.61-2.83) | 0.072  **0.032**  0.49 |
| Stage 1  1+2  2  2+3  3  3+4 | 170  254  84  167  83  88 | 1.62（0.96-2.74）  0.69（0.45-1.06）  0.44（0.24-0.82）  0.49（0.33-0.74）  0.45（0.24-0.82）  0.45（0.25-0.81） | 0.071  0.088  **0.0083**  **0.00043**  **0.0074**  **0.0066** | 167  249  82  163  81  84 | 3.18(0.74-13.74)  0.67(0.34-1.34)  0.33(0.1-1.08)  0.38(0.21-0.7)  0.36(0.17-0.78)  0.37(0.18-0.79) | 0.1  0.26  0.054  **0.0013**  **0.007**  **0.0075** |

^*^ The sample numbers with some clinical characteristics were too low for meaningful analysis, such as Black or Africa American, Grade 4, macro invasion, etc. N: sample size.

**Table 5. The survival analysis of *SRGN* expression associated with infiltrating cells in LIHC by TIMER2.0**

| Cells in TME | Low SRGN  expression  Hazard ratio | | *p*-value | | High SRGN  expression  Hazard ratio | | *p*-value | |  |
| --- | --- | --- | --- | --- | --- | --- | --- | --- | --- |
| T cell CD8+  T cell CD4+ memory resting  Hematopoietic stem cell  MDSC  Endothelial cell  Monocyte(MCPcounter) | | 0.658  0.847  0.834  1.41  0.565  1.65 | | 0.139  0.584  0.551  0.313  0.0566  0.105 | | 0.53  0.501  0.379  2.78  0.364  2.75 | | **0.0448**  **0.0383**  **0.00868**  **0.00668**  **0.00638**  **0.025** | |
| Macrophage  Macrophage M1  Macrophage 0  Macrophage 2 | | 1.82  1.19  1.86  1.99 | | **0.0432**  0.558  **0.0356**  **0.0366** | | 1.58  3.09  1.59  1.25 | | 0.198  **0.00633**  0.159  0.568 | |
| T cell CD4+ central memory  T cell CD4+ Th2  Tregs  Monocyte(XCELL)  Myloid dendrtic cell  Myloid dendrtic cell resting | | 0.742  1.22  0.873  1.19  0.924  1.34 | | 0.305  0.498  0.659  0.599  0.796  0.283 | | 0.874  1.83  0.671  1.95  3.28  1.05 | | 0.694  0.0945  0.249  0.0811  0.0599  0.871 | |

TME: tumor microenvironment
